# Pectolinarigenin regulates the tumor-associated proteins in AGS-xenograft BALB/c nude mice

**DOI:** 10.1007/s11033-023-09046-4

**Published:** 2024-02-16

**Authors:** Ho Jeong Lee, Young Sang Kwon, Ju Hong Lee, Yeon Gyu Moon, Jungil Choi, Moonjung Hyun, Tae Kil Tak, Je-Hein Kim, Jeong Doo Heo

**Affiliations:** 1 Gyeongnam Bio-Health Research Support Center, Gyeongnam Branch Institute, Korea Institute of Toxicology (KIT), 17 Jeigok-gil, Jinju, 52834 Republic of Korea; 2 Environmental Safety Assessment Center, Gyeongnam Branch Institute, Korea Institute of Toxicology (KIT), 17 Jeigok-gil, Jinju, 52834 Republic of Korea

**Keywords:** Pectolinarigenin, AGS-xenograft, Proteomics, Tumor suppress, PI3K/AKT/mTOR

## Abstract

**Background:**

Pectolinarigenin (PEC) is a flavone extracted from *Cirsium*, and because it has anti-inflammatory properties, anti-cancer research is also being conducted. The objective of this work was to find out if PEC is involved in tumor control and which pathways it regulates in vivo and in vitro.

**Methods:**

AGS cell lines were xenografted into BALB/c nude mice to create tumors, and PEC was administered intraperitoneally to see if it was involved in tumor control. Once animal testing was completed, tumor proteins were isolated and identified using LC–MS analysis, and gene ontology of the found proteins was performed.

**Results:**

Body weight and hematological measurements on the xenograft mice model demonstrated that PEC was not harmful to non-cancerous cells. We found 582 proteins in tumor tissue linked to biological reactions such as carcinogenesis and cell death signaling. PEC regulated 6 out of 582 proteins in vivo and in vitro in the same way.

**Conclusion:**

Our findings suggested that PEC therapy may inhibit tumor development in gastric cancer (GC), and proteomic research gives fundamental information about proteins that may have great promise as new therapeutic targets in GC.

**Supplementary Information:**

The online version contains supplementary material available at 10.1007/s11033-023-09046-4.

## Introduction

Cancer is well known around the world as a disease that endangers human life, and research is being performed to produce new medications for treating cancer and improving environmental conditions. Cancer, often known as a malignant tumor, is a condition in which the cell cycle and cell division are uncontrolled [1]. Gastric cancer (GC) has the second-highest global incidence and is common throughout East Asia, particularly Korea [2]. According to recent research, more than half of GC cases are linked to alcohol intake, smoking, infections, food, and obesity [3, 4]. Although different research are being undertaken to uncover potential therapies for GC, early identification remains difficult, and it has a high recurrence rate following surgery; GC is also one of the most difficult malignancies to cure due to chemotherapy side effects [5]. Anticancer medications with low side effects that do not impact healthy cells are required to overcome these obstacles. The development of medicines based on natural ingredients has gained popularity. We previously investigated the efficacy natural flavonoid active components in killing cancer cells in different malignancies, and we recently investigated the efficacy of pectolinarigenin (PEC) in GC cell lines.

Flavonoids are the most prevalent and widely eaten polyphenolic compounds found in plants. Flavonoids contain anti-inflammatory and antioxidant properties and have a variety of impacts such as inflammatory bowel disease (IBD) and cardiovascular disease [6–9]. They are also being intensively researched in various cancer cell types. In gastrointestinal cancer, flavonoids influence cancer via pathways such as Wnt/β-catenin, PI3K/AKT/mTOR, AMP-activated protein kinase (AMPK), mitogen-activated protein kinase (MAPK), and nuclear factor-kappa B (NF-kB) [10]. Furthermore, flavonoids influence cancer through a variety of mechanisms in different cancer cells, including oxidative stress, Ras, and STAT3 [6].PEC is a flavonoid mainly found in *Cirsium isolates* [11]. In our previous study, we confirmed that PEC is abundant in *Citrus* fruits such as *Citrus platymamma* [12]. PEC has been shown to have anti-inflammatory and modulating effects on various cancers [12–14]. We previously confirmed that PEC induces G2/M phase arrest along with autophagic and apoptotic cell death through the PI3K/AKT/mTOR pathway in both AGS and MKN28 human GC cells [15]. In addition, we found that PEC regulated the expression of LRSAM1, DDX4, PK3CB, and CIP2A in both AGS and MKN28 cells via comparative proteomic analysis [15, 16].

Proteomic analysis is developing as an essential analytical tool for identifying that may be used to diagnose a variety of disorders, including cancer [17]. To enhance patient outcomes, early detection of illness, including GC, requires research in molecular biology and biomarker development. The molecular nature of anti-cancer medicines, including their impact on cancer signaling and therapeutic response in tumor cells, is revealed through proteomic research. Furthermore, biomarkers discovered by proteomic analysis may be valuable in the development of medicinal drugs [18, 19].

To test the effectiveness of PEC in vivo, we used GC cell xenografts in BALB/c nude mice to induce tumor growth, and then used liquid chromatography-tandem mass spectrometry (LC–MS/MS) to identify tumor-associated proteins. Proteins with changed abundance following PEC therapy can be examined in future investigations of PEC’s anti-cancer efficacy.

## Materials and methods

### Chemicals and reagents

The AGS human gastric cancer cells were purchased from the American Type Culture Collection (ATCC) (Rockville, MD, USA). RPMI-1640 medium, fetal bovine serum (FBS), and antibiotics (Penicillin/Streptomycin) were purchased from Gibco; Thermo Fisher Scientific, Inc. (Waltham, MA, USA). Matrigel® Matrix was purchased from Corning (NY, USA). 5-Fluorouracil (5-FU) and dimethyl sulfoxide (DMSO) were purchased from Sigma-Aldrich (St. Louis, MO, USA).

### Animal

The female BALB/c-nude mouse (5 weeks old) was obtained from Koatech (Pyeong-taek, Korea). The feed and water were given *ad livitum*. All animals have resided in a room with a temperature of 20–27 °C, relative humidity 40–60%, approximately 12 h light/12 h dark cycle, and ventilation 10–20 times/hour. Before entering the experiment, the animals were acclimatized to the animal laboratory environment for 1 week. All animal care and experimental studies were conducted according to the guidelines. This animal study was scrutinized and assessed by the Korea Institute of Toxicology, Gyeongnam Branch Institute Institutional Animal Care and Use Committee (2008-0006).

### Mice xenograft models

For the tumor xenograft mice model, 1 × 10^6^ AGS cells and matrigel were mixed 1:1 ratio and were injected subcutaneously administration with in the nape of BALB/c-nude mice. After the AGS cells were injected, the tumor size was measured and observed until the tumor volume grew to about 100 mm^3^. After growing to 100 mm^3^ of the tumor, mice were divided randomly into four groups (n = 6 in each group) and administrated intraperitoneal injected with 5-FU 10 mg/kg, PEC 25 mg/kg, 50 mg/kg, or vehicle (5% DMSO) three times a week for 3 weeks. The tumor growth and body weight were measured twice a week, and the tumor volume was calculated by measuring the length (a, mm) and width (b, mm) with a caliper as follows (Tumor volume (mm^3^) = a × b^2^ × 0.5). After 3 weeks treated mice were sacrificed for dissecting tumors under anesthesia by CO_2_. Before dissecting the tumors, blood was collected from each group, and put into EDTA-tube and SST for hematological and biochemical blood analysis.

### Extraction of tumor proteins

Whole proteins were extracted from all groups of the tumor by protein extraction buffer (0.1 M phenylmethylsulfonyl fluoride (PMSF), 2% β-mercaptoethanol). After vortex and centrifuge, the supernatant transfers to a new tube and add 0.1 M ammonium acetate. Re-moved the supernatant, and add 80% cold-acetone for washing, and did speed-vacuum to dry the pellet. The pellet dissolved in lysis buffer (7 M urea, 2 M thiourea, 4% CHAPS, 1 mM PMSF, and 50 mM dithiothreitol (DTT)). The total soluble protein concentration was determined by the 2-D Quant kit (Sigma-Aldrich, St. Louis, MO, USA) in accordance with the manufacturer’s protocol.

### One-dimensional SDS-PAGE

To prepare one-dimensional electrophoretic protein samples., we added denaturing sample buffer (0.5 M Tris-HCl pH 5.5, 10% sodium dodecyl sulfate (SDS), 20% glycerol, 1% bromophenol blue, and 0.2% DTT) and heated at 95 °C for 5 min. Samples were given an equal amount of 50 µg to each well, and sodium dodecyl sulfate-polyacrylamide gel electrophoresis (SDS-PAGE) was performed on 12% gel. The gels were stained with Coomassie Blue and destained with distilled water to stain the specific protein.

### Trypsin digestion and sample preparation

The protein bands were fractionated for each lane, and then divided into 3–5 rows and sliced. The sliced gels were purified using the acetonitrile (ACN) and were equilibrated with 10 mM DTT/0.1 M ammonium bicarbonate and 55 mM iodoacetamide (IAA)/0.1 M ammonium bicarbonate. The gel pieces were rehydrated using the ACN and speed-vacuum for dry and put in 50 µL digestion buffer [25 mM ammonium bicarbonate, 0.1% octyl β-D-glucopyranoside (OGP), and 20 µg/vial trypsin (Promega, Madison, MI, USA)]. The chopped gels were placed in a siliconized tube containing 100 µL non-trypsin digestion buffer for enzymatic cleavage and incubated at 37 °C overnight. After centrifugation to obtain the active ingredient, the supernatant was transferred to a new tube and dried with a speed-vacuum drier. The dried pellet was dissolved in 50% ACN, 0.1% trifluoroacetic acid (TFA) solution.

The lysates were dried with a speed-vacuum drier for 3 h and the dried pellet was dissolved in 1% TFA solution. The peptide lysate was extracted with Oasis PRiME HLB SPE cartridges coupled to the vacuum manifold. 100% ACN and 1% TFA have sequentially flowed through the cartridge, and loaded the peptide sample onto a cartridge. After loading the sample, add 0.1% TFA and 50% ACN to the cartridge and elute the peptide into a new tube using a syringe. The peptide was dried with speed-vacuum overnight and the pellet dissolved in 0.1% formic acid (FA) solution.

### LC–MS/MS analysis

The quantified protein samples were analyzed using invented by an Ultimate 3000 liquid chromatography system coupled with a Q-Exactive Hybrid Quadrupole-Orbitrap mass spectrometer (Thermo Scientific, Bremen, Germany). MS spectra were acquired at a resolution of 70,000 within a mass range of 400–2,000 m/z. Samples were loaded in a trapping cartridge (Acclaim PepMap C18 100 Å, 5 mm × 300 μm i.d., 160,454, Thermo Scientific) and checked at the rate of 3 µL/min in a mobile phase of 5% ACN, 0.1% FA. After loading for 5 min, the trap column was changed in-line to a 15 cm by 75 μm inner diameter EASY-Spray column (ES800, PepMap RSLC, C18, 3 μm, Thermo Scientific, Bremen, Ger-many) at 300 nL/min. Separation was made by mixing 0.1% FA and 80% ACN, and LC gradient was carried out under the following conditions; 0–5% ACN in 10 min, 10–40% ACN in 60 min, stay at 95% ACN for 15 min, and 95-% ACN in 15 min. Separation was generated by mixing A: 0.1% FA and B: 80% ACN, using the following LC gradient: 0–5% B in 10 min, 10–40% B in 60 min, and stay at 95% B for 15 min. The data obtained after separation were searched in the human Swissprot database using the Sequest HT (Thermo Fisher Scientific) with Proteome Discoverer software version 2.3(Thermo Fisher Scientific) and quantified with a label-free quantification approach. Precursor and fragment mass tolerances were set at 10 ppm and 0.02 Da, respectively. Trypsin was set as the enzyme, and up to two missed cleavages were permitted. Peptides were filtered with FDR at 1% with a Benjamini–Hochberg correction.

### Gene ontology (GO) and protein–protein interaction

Gene ontology (GO) studies can reveal the information of expressed proteins including the biological process, cellular components, molecular functions, and pathways. The profile of the expressed protein was analyzed with GENECODIS database version 4.0 (http://genecodis.genyo.es), WebGestalt (WEB-based Gene SeT AnaLysis Toolkit) (http://webgestalt.org), and PANTHER (protein analysis through evolutionary relationships) database version 16.0 (http://pantherdb.org). Also, we used STRING (Search Tool for the Retrieval of Interacting Genes) database version 11.5 (http://string-db.org) to investigate potential protein-protein interactions of selected genes.

### RT-qPCR

Total RNA was extracted using Trizol reagent (Invitrogen, MA, USA) according to the manufacturer’s instructions from AGS cell lines treated with or without PEC at indicated concentrations. The RNAs were quantified using a Nano Drop spectrophotometer. RNA was reverse-transcripted at 42 °C for 15 min using cDNA synthesis kits (Meridian bioscience, OH, USA). The cDNA was subsequently amplified by PCR using the SensiFAST SYBR & Fluorescein Kit (Meridian bioscience, OH, USA) according to the manufacturer’s recommendation in a Thermal Cycler Dice® Real Time System III (TaKaRa Bio, Japan). Relative fold levels were determined using GAPDH genes as normalizer control. The primers (sequences: for ANXA11, F: 5ʹ-AACATGCCCAACCTGTACCC-3ʹ and R: 5ʹ-ATAGGGAGGAACAGGCTGCT-3ʹ; for CAMK2D, F: 5ʹ-CTGCCGTCTTTTGAAGCACC-3ʹ and R: 5ʹ-TGACTGGCATCAGCTTCACT-3ʹ; for CTSD, F: 5ʹ-GTGGAGGACCTGATTGCCAA-3ʹ and R: 5ʹ-ACTGGGCGTCCATGTAGTTC-3ʹ; for EIF4E, F: 5ʹ-TGCGGCTGATCTCCAAGTTT-3ʹ and R: 5ʹ-AAGCGATCGAGGTCACTTCG-3ʹ; for MAPK1, F: 5ʹ-ACCTACTGCCAGAGAACCCT-3ʹ and R: 5ʹ-TCGATGGTTGGTGCTCGAAT-3ʹ; for RHOA, F: 5ʹ-TCGAGGTGGATGGAAAGCAG-3ʹ and R: 5ʹ-TCAGGGCTGTCGATGGAAAA-3ʹ; for GAPDH, F: 5ʹ-GGAGCGAGATCCCTCCAAAAT-3ʹ and R: 5ʹ-GGCTGTTGTCATACTTCTCATG-3ʹ) were purchased from Bioneer (Seoul, Korea).

### Statistical analysis

All dissimilarities between the groups were investigated for statistical significance with one-way ANOVA test using GraphPad Prism version 5.01 (San Diego, CA, USA) was utilized for data analysis. The data were expressed as mean ± standard deviation (SD) of at least three independent experiments. A value of p < 0.05 was considered significant.

## Results

### Suppressed tumor growth in AGS-xenograft mouse model

**Fig. 1:**
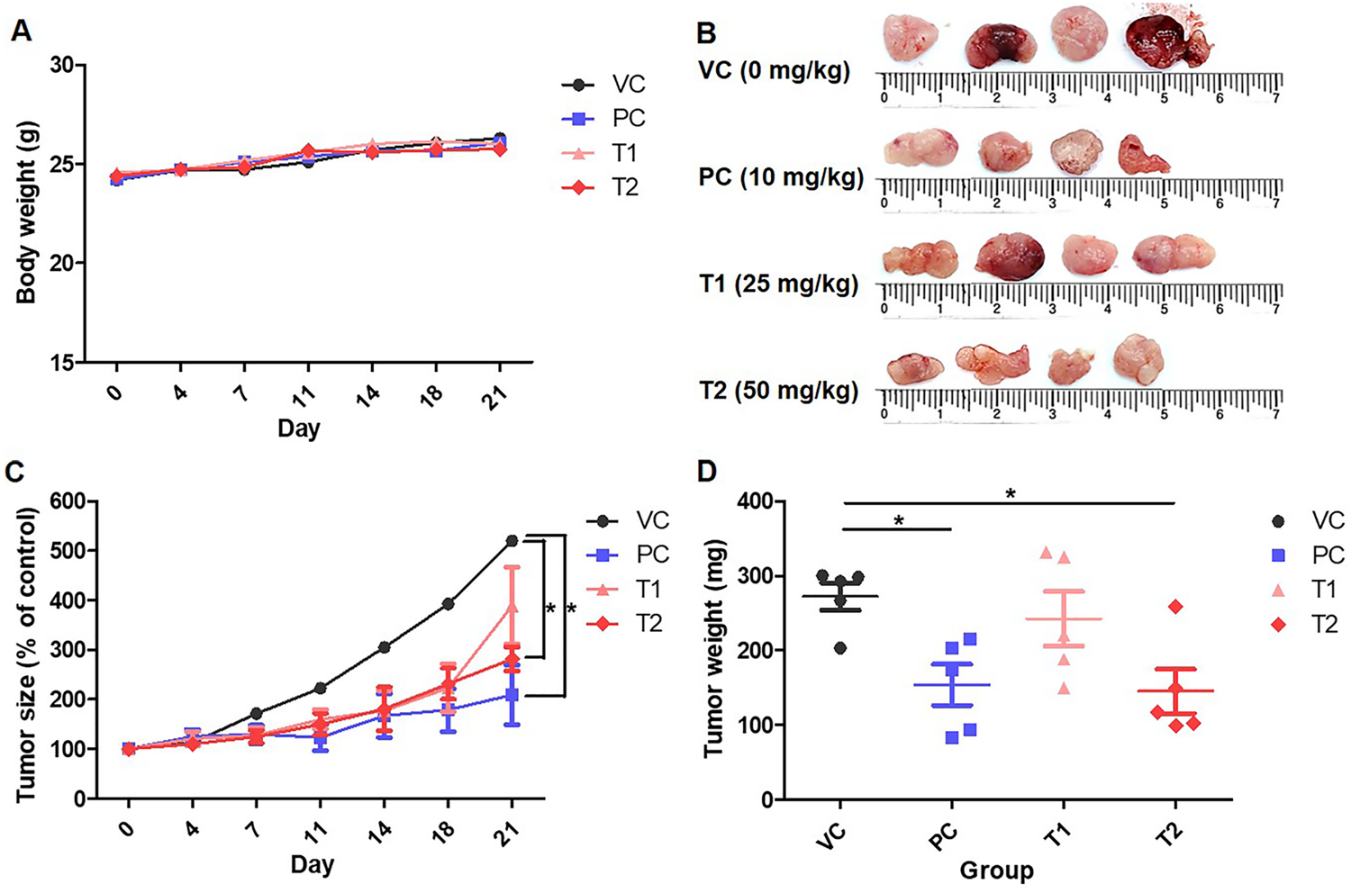
Inhibitory effects of PEC on the growth of AGS-xenograft BALB/c nude mice. **A** Body weights of the mice from VC, PC, T1, and T2 groups for 3 weeks. **B** Presentative photographs of the tumor from VC, PC, T1, and T2 groups after final sacrifice. **C** Tumor size and **D** tumor weight of the mice from VC, PC, T1, and T2 groups for 3 weeks and final day. Values given are expressed as mean ± standard deviation (SD). The asterisk (*) indicates statistical significance (p < 0.05) compared with the control group

AGS cells were implanted subcutaneously into the nape of BALB/c nude mice to construct an AGS-xenograft mouse model to examine the tumor-suppressive impact of PEC. PEC concentrations of 25 and 50 mg/kg were employed and administered three times a week for 3 weeks following tumor growth, and subsequent changes in tumor growth, body weight, and tumor weight were observed three times a week for 3 weeks. As a positive control, 5-FU was given at a dosage of 10 mg/kg to AGS-xenograft BALB/c nude mice. PEC therapy reduced tumor growth and tumor weight in a dose-dependent manner (Fig. 1A and B, and 1C). However, body weight did not significantly change in any of the groups (Fig. 1D; Table 1). Blood was obtained after the sacrifice to undertake biochemical and hematological assays. There were no significant differences between the groups (Tables 2 and 3).

**Table 1:** Inhibition effect of pectolinarigenin on xenograft tumor growth in Balb/c nude mice

Group (n = 6)	Dose (mg/kg)	Mean tumor volume, V/mm^3^	Mean tumor weight, m/mg	Inhibitor rate (%)
VC	–	659.14 ± 249.27	276.26 ± 150.19	–
PC	10	237.73 ± 119.67	151.21 ± 121.13	63.93
T1	25	462.09 ± 214.34	216.30 ± 107.55	29.89
T2	50	321.78 ± 102.81	181.30 ± 123.04	51.18

**Table 2:** Hematological parameter of AGS cell xenograft Balb/c nude mice

Prarameter	Unit	VC	PC	T1	T2
WBC	$\times$10^3^cells/µL	3.3 ± 1.3	4.2 ± 2.9	3.1 ± 1.0	3.8 ± 1.8
RBC	$\times$10^6^cells/µL	8.3 ± 0.6	8.3 ± 0.8	8.2 ± 0.7	8.6 ± 0.4
HGB	g/dL	13.5 ± 0.8	13.3 ± 0.9	13.61.2	13.7 ± 0.9
HCT	%	47.0 ± 4.0	46.2 ± 3.6	46.9 ± 5.1	48.6 ± 3.1
MCV	fL	56.4 ± 1.5	55.5 ± 3.8	56.9 ± 1.9	56.8 ± 1.4
MCH	pg	16.3 ± 0.4	16.0 ± 1.0	16.5 ± 0.3	16.0 ± 0.4
MCHC	g/dL	28.8 ± 0.7	28.8 ± 1.1	29.0 ± 0.8	28.3 ± 0.7
RDW	%	13.3 ± 0.8	15.1 ± 3.4	12.8 ± 0.7	13.0 ± 0.5
PLT	$\times$10^3^cells/µL	712.0 ± 78.1	670.0 ± 80.7	660.0 ± 84.4	682.5 ± 76.6
Neutrophils	$\times$10^3^cells/µL	18.9 ± 7.1	17.7 ± 5.6	13.9 ± 3.7	16.1 ± 3.9
Lymphocytes	$\times$10^3^cells/µL	75.8 ± 7.0	77.6 ± 7.5	80.8 ± 3.4	79.6 ± 4.4
Monocytes	$\times$10^3^cells/µL	0.7 ± 0.1	2.2 ± 2.5	3.3 ± 2.5	1.6 ± 1.5
Eosinophils	$\times$10^3^cells/µL	2.7 ± 2.5	1.2 ± 0.6	1.1 ± 0.5	1.6 ± 0.6
Basophils	$\times$10^3^cells/µL	0.1 ± 0.1	0.1 ± 0.1	0.1 ± 0.1	0.1 ± 0.1

*WBC* Leukocyte count, *RBC* Erythrocyte count, *HGB* Hemoglobin, *HCT* Hematocrit, *MCV* Mean corpuscular volume, *MCH* Mean corpuscular hemoglobin, *MCHC* Mean corpuscular hemoglobin concentration, *RDW* Red cell distribution width, *PLT* Platelet

**Table 3:** Blood chemistry parameter of AGS cell xenograft Ballb/c nude mice

Prarameter	Unit	VC	PC	T1	T2
AST	U/L	75.2 ± 15.1	70.3 ± 12.6	79.7 ± 28.1	72.2 ± 11.0
ALT	U/L	19.3 ± 4.0	17.3 ± 3.3	20.2 ± 3.2	19.0 ± 6.2
Urea	mg/dL	20.8 ± 2.1	20.0 ± 4.8	21.2 ± 3.5	23.8 ± 6.2
Creatinine	mg/dL	0.2 ± 0.1	0.2 ± 0.1	0.2 ± 0.1	0.2 ± 0.1
TG	mg/dL	64.5 ± 24.6	77.0 ± 23.0	104.2 ± 29.9	77.3 ± 18.5
TC	mg/dL	129.0 ± 15.5	124.3 ± 14.0	122.3 ± 15.9	148.3 ± 12.4

*AST* Aspartate aminotransferase, *ALT* Alanine aminotransferase, *TG* Triglyceride, *TC* Total cholesterol

### Identification of differentially expressed proteins by LC–MS/MS

We retrieved the tumor from each group, such as the vehicle control (VC), positive control as 5-FU 10 mg/kg (PC), PEC 25 mg/kg (T1), and PEC 50 mg/kg (T2), and extracted the protein in the tumor. We used the one-dimension electrophoresis (1-DE) to separate the gel samples from each group. Preprocessing raw serial mass spectrometry data using Proteome DiscoverTM software (version 3.0) and the UniProt database (Supplement 1) yielded protein expression lists for the full protein profile between the VC, PC, T1, and T2 samples. On LC-MS/MS analysis of AGS-xenograft tumors, a total of 582 differentially expressed proteins from each group were discovered. PEC treatment indicated that 168 proteins were elevated and 414 proteins were downregulated among the identified proteins. As a consequence of filtering with fold change ≥ 1.5 and p-value < 0.05, the protein identification is trustworthy.

### Gene ontology analysis

**Fig. 2:**
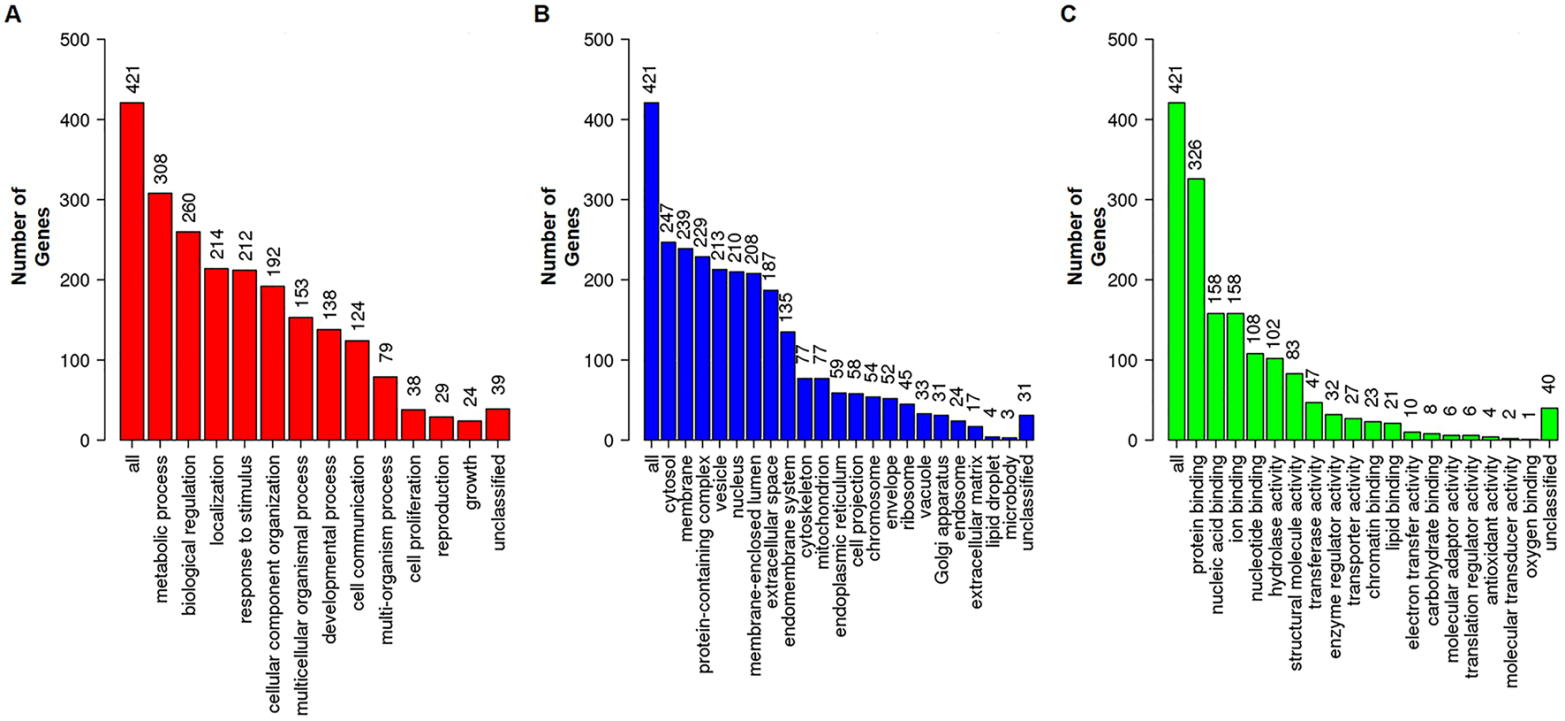
Cellular function of tumor proteins on AGS-xenograft BALB/c nude mice due to PEC treatment. **A** Biological processes, **B** cellular components, and **C** molecular functions in tumor proteins on AGS-xenograft mice model. Analysis was performed using WebGestalt

We utilized WebGestalt software to generate the data displayed in Fig. 2A–C to confirm the gene ontology of the proteins discovered through LC–MS/MS analysis. The GO results identified 469 proteins; the three strongest associations with biological process were metabolic process (GO:0008152), biological regulation (GO:0065007), and response to stimulus (GO:0050896). Proteins in the cellular component were associated with the cytosol (GO:0005829), membrane (GO:0016020), and protein-containing complex (GO:0032991). The molecular functions of the differentially expressed proteins included protein binding (GO:0005515), ion binding (GO:0043167), and nucleic acid binding (GO:0003676).

### Pathway analysis of the differentially expressed proteins

**Fig. 3:**
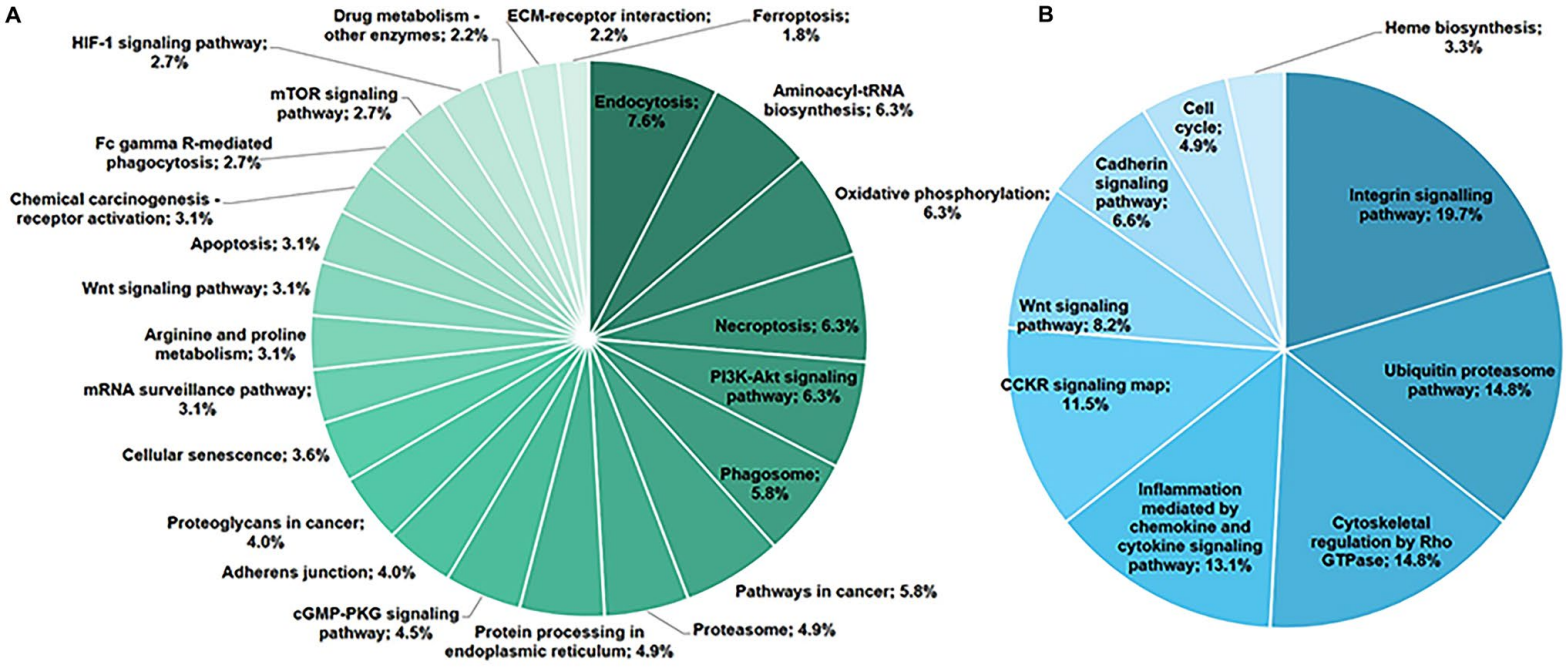
Pathway of tumor proteins on AGS-xenograft BALB/c nude mice due to PEC treatment. The pie charts show **A** the KEGG pathway and **B** the PANTHER pathway associated with tumor proteins on the AGS-xenograft mice model. Analysis was performed using GeneCodis and PANTHER

Using the GeneCodis database (https://genecodis.genyo.es), we discovered the pathways associated with differentially expressed proteins. GeneCodis contains two types of pathway analyses: KEGG and PANTHER. The 582 proteins were uploaded to the GeneCodis database for pathway enrichment analysis, which indicated 24 and 12 significant pathways respectively, based on KEGG and PANTHER analysis, as shown in Fig. 3; Table 4, and Table 5. Tables 4 and 5 show the detailed individual pathways with protein distribution in the AGS-xenograft mice model injected with or without PEC.

**Table 4:** KEGG pathway analysis of pectolinarigenin on AGS-xenograft Balb/c nude mice

Pathways	Annotaion ID	No. of genes	p-value	Genes
Aminoacyl-tRNA biosynthesis	hsa00970	14	6.61E-25	LARS2, GARS1, HARS2, WARS1, EPRS1, AARS1, MARS1, QARS1, IARS2, FARSB, IARS1, FARSA, HARS1, DARS1
Oxidative phosphorylation	hsa00190	14	4.13E-21	COX4I1, COX5A, PPA1, NDUFS3, UQCRC1, ATP5MF, NDUFA5, NDUFA4, NDUFV2, NDUFS7, COX6C, NDUFA10, NDUFA13, ATP6V1A
Proteasome	hsa03050	11	1.89E-18	PSMD3, PSMC6, PSMC2, PSMD13, PSMC3, PSMD2, PSMC4, PSMD1, PSMD7, PSME2, PSMD8
Endocytosis	hsa04144	17	2.78E-17	VPS35, PDCD6IP, ARPC2, RAB8A, ARPC4, ARF4, RHOA, KIF5B, CAPZA1, RAB5C, ACTR3, ARPC3, CHMP4A, VPS37B, RAB5A, SNX2, CHMP4B
Necroptosis	hsa04217	14	8.70E-14	HSP90AB1, VDAC1, VDAC2, H2AC21, SLC25A6, HSP90AA1, PYGM, CAPN1, CHMP4A, CHMP4B, HMGB1, PARP1, CAMK2D, PPID
Phagosome	hsa04145	13	7.19E-13	TUBA1C, CORO1A, TUBB3, ACTB, TUBA1B, DYNC1LI2, RAB5C, RAB5A, C3, TUBB2A, DYNC1H1, TUBB4B, ATP6V1A
Protein processing in endoplasmic reticulum	hsa04141	11	6.16E-12	HSP90AB1, HSP90B1, SEC31A, DDOST, HSP90AA1, CKAP4, CAPN1, ERO1A, SSR4, SEC24C, VCP
Adherens junction	hsa04520	9	2.49E-09	ACTN4, VCL, ACTB, RHOA, MAPK1, CSNK2B, TJP1, YES1, CSNK2A2
mRNA surveillance pathway	hsa03015	7	2.51E-09	PYM1, UPF1, ETF1, PPP2R1A, PPP2R2A, PPP1CB, ALYREF
Arginine and proline metabolism	hsa00330	7	7.54E-09	PYCR1, CKM, ALDH1B1, GOT2, SRM, MAOB, CKMT1A
PI3K-Akt signaling pathway	hsa04151	14	2.73E-08	GYS1, HSP90AB1, HSP90B1, LAMB1, HSP90AA1, MAPK1, GNB1, LAMC1, PPP2R1A, PPP2R2A, RPS6, COL1A1, LAMA1, EIF4E
cGMP-PKG signaling pathway	hsa04022	10	8.99E-08	VDAC1, VDAC2, PPP1R12A, SLC25A6, RHOA, MAPK1, MYH6, VASP, PPP1CB, ATP1A1
Drug metabolism - other enzymes	hsa00983	5	2.08E-06	GSTT1, CES1, HPRT1, GMPS, TPMT
Wnt signaling pathway	hsa04310	7	4.33E-06	RHOA, CSNK2B, CACYBP, RUVBL1, CSNK2A2, CAMK2D, CTBP2
Cellular senescence	hsa04218	8	6.29E-06	VDAC1, VDAC2, SLC25A6, MAPK1, CAPN1, MRE11, PPP1CB, PPID
Proteoglycans in cancer	hsa05205	9	7.53E-06	PPP1R12A, ACTB, RHOA, MAPK1, LUM, RPS6, PPP1CB, COL1A1, CAMK2D
Fc gamma R-mediated phagocytosis	hsa04666	6	1.83E-05	ARPC2, ARPC4, MAPK1, VASP, ACTR3, ARPC3
Apoptosis	hsa04210	7	3.45E-05	TUBA1C, ACTB, MAPK1, TUBA1B, CAPN1, CTSD, PARP1
Pathways in cancer	hsa05200	13	4.12E-05	HSP90AB1, HSP90B1, GSTT1, LAMB1, RHOA, HSP90AA1, MAPK1, GNB1, LAMC1, LAMA1, CAMK2D, CTBP2, EML4
ECM-receptor interaction	hsa04512	5	4.59E-05	AGRN, LAMB1, LAMC1, COL1A1, LAMA1
mTOR signaling pathway	hsa04150	6	6.43E-05	RHOA, MAPK1, SLC3A2, RPS6, EIF4E, ATP6V1A
HIF-1 signaling pathway	hsa04066	6	6.92E-05	MAPK1, PDHA1, PGK1, RPS6, EIF4E, CAMK2D
Ferroptosis	hsa04216	4	8.11E-05	CP, VDAC2, PCBP1, SLC3A2
Chemical carcinogenesis-receptor activation	hsa05207	7	8.90E-05	KPNB1, HSP90AB1, HSP90B1, GSTT1, HSP90AA1, MAPK1, KPNA4

**Table 5:** PANTHER pathway analysis of pectolinarigenin on AGS-xenograft Balb/c nude mice

Pathways	Annotaion ID	No. of Genes	p-value	Genes
Ubiquitin proteasome pathway	P00060	9	1.75E-20	UBE2N, PSMD13, PSMD2, PSMC4, UBA1, PSMD1, UBE2K, UBA2, PSMD7
Integrin signalling pathway	P00034	12	2.10E-19	ACTN4, ARPC2, TLN1, VCL, LAMB1, ACTB, RHOA, VASP, LAMC1, ARPC3, COL1A1, LAMA1
Cytoskeletal regulation by Rho GTPase	P00016	9	1.60E-17	ARPC2, TUBB3, ARPC4, ACTB, MYH6, VASP, ARPC3, TUBB2A, TUBB4B
Inflammation mediated by chemokine and cytokine signaling pathway	P00031	8	8.14E-15	ARPC2, ARPC4, ACTB, RHOA, MAPK1, MYH6, ARPC3, CAMK2D
CCKR signaling map	P06959	7	2.43E-10	ELAVL1, RHOA, MAPK1, GNB1, RPS6, EIF4E, YES1
Cadherin signaling pathway	P00012	4	1.64E-08	ACTB, CSNK2B, YES1, CSNK2A2
Cell cycle	P00013	3	8.92E-08	PSMD3, PSMD13, PSMD7
Wnt signaling pathway	P00057	5	2.34E-07	GNB1, CSNK2B, MYH6, CSNK2A2, CTBP2
Heme biosynthesis	P02746	2	8.97E-07	EPRS1, QARS1
5-hydroxytryptamine degredation	P04372	2	1.71E-06	ALDH1B1, MAOB

### Gene expressions and protein–protein interactions in PI3K/AKT/mTOR signaling pathway

**Fig. 4:**
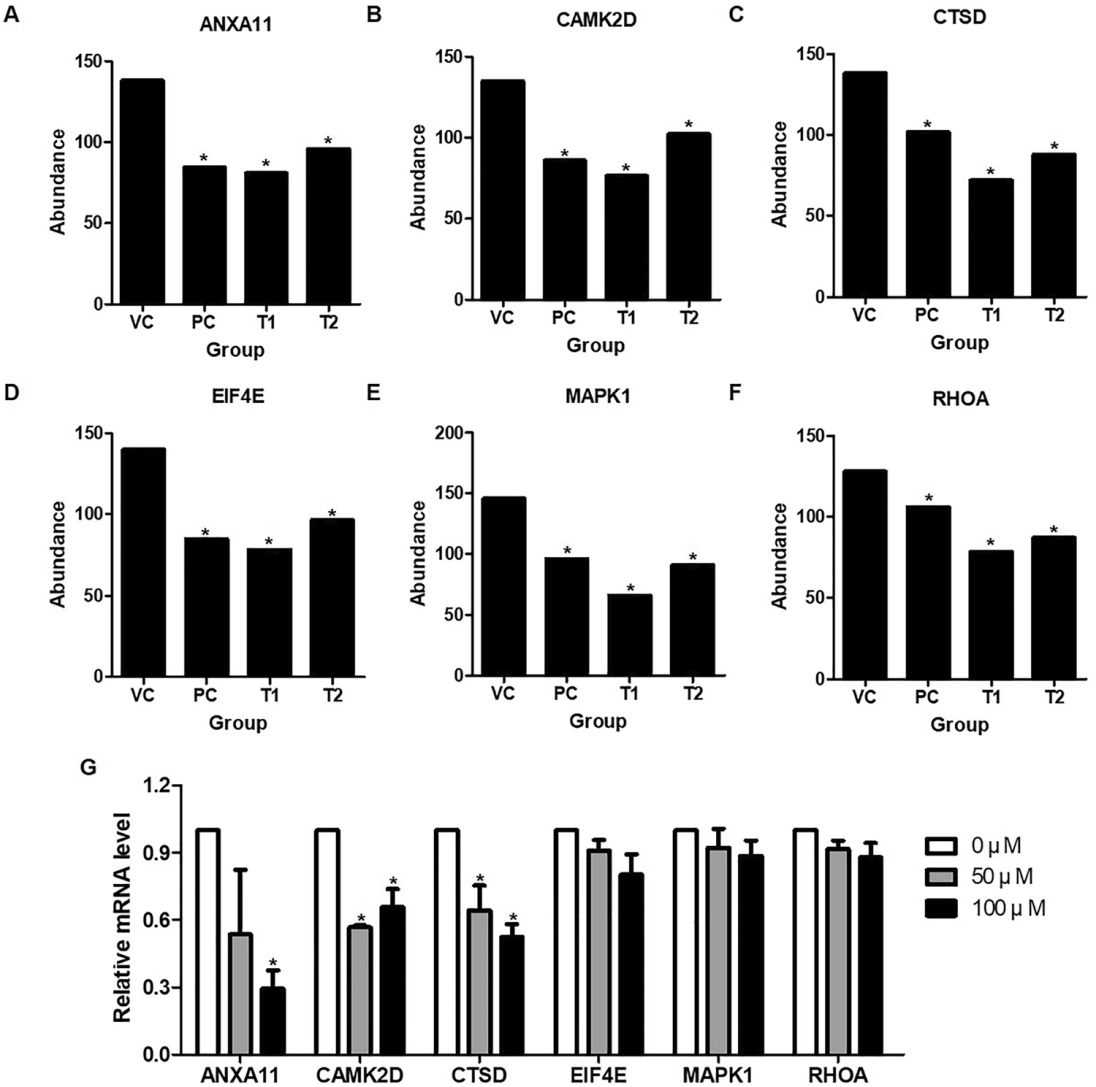
Effects of PEC on gene expression of tumor on AGS-xenograft BALB/c nude mice. The graphs show the comparison between the average normalized volume of each gene **A** ANXA11, **B** CAMK2D, **C** CTSD, **D** EIF4E, **E** MAPK1, **F** RHOA from the VC, PC, T1, and T2 groups. The data of the differentially expressed proteins were quantified by Proproteins analysis software. **G** The result of confirming the mRNA expression of 6 factors in AGS cells treated with or without PEC by RT-qPCR. The bar indicates normalized to GAPDH. The volumes are expressed as a mean of normalized gene volume ± standard deviation (SD) of the normalized volume of the total genes (*p < 0.05 vs. VC)

**Fig. 5:**
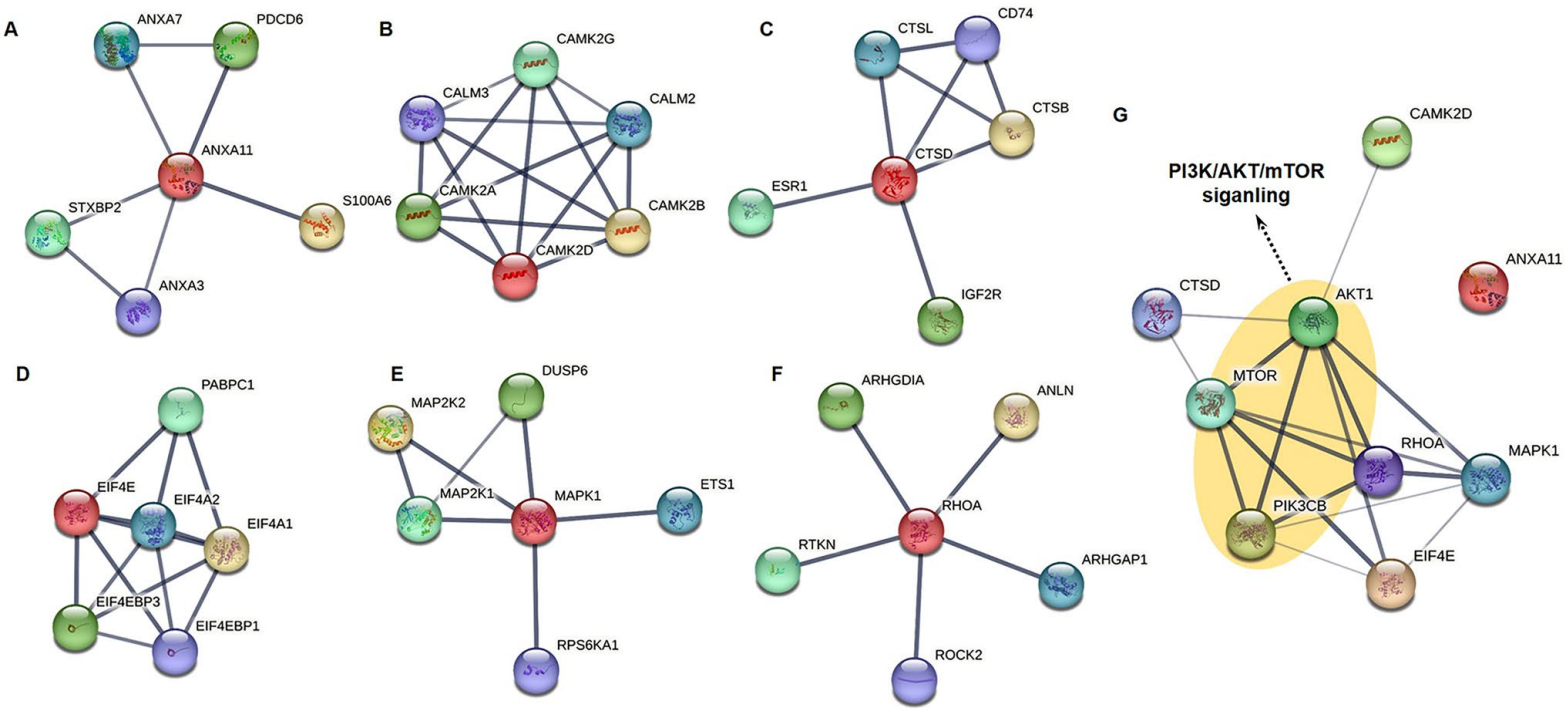
Relationship between major genes and the PI3K/AKT/mTOR signaling pathway. The image shows the protein-protein interaction network of each gene (**A**–**F**), and its relationship with the PI3K/AKT/mTOR signaling pathway (**G**). Analysis was performed by STRING data base

We analyzed genes that are frequently detected in both the KEGG and PANTHER pathways and those commonly involved in pathways associated with cancer cell death, such as apoptosis. We selected 6 genes, including *ANXA11*, *CAMK2D*, *CTSD*, *EIF4E*, *MAPK1*, and *RHOA* which were confirmed to be downregulated by PEC (Fig. 4). As a result of confirming the mRNA expression of the 6 major factors in vitro, it was confirmed that the decrease was similar to the result of tumor analysis by PEC treatment (Fig. 4G). *CTSD*, MAPK1, and *RHOA* showed greater downregulation in the T2 group than in the PC group. As a result of confirming protein-protein interactions of 6 major factors in STRING, ANXA11 interacted with PDCD6, ANXA7, STXBP2, ANXA3, and S100A6; CAMK2D interacted with CAMK2A, CAMK2B, CAMK2G, CALM2, and CALM3; CTSD interacted with CTSB, CTSL, CD74, ESR1, and IGF2R; EIF4E interacted with EIF4A1, EIF4A2, EIF4EBP1, EIF4eBP3, and PABPC1; MAPK1 interacted with MAP2K1, MAP2K2, DUSP6, ETS1, and RPS6KA1; RHOA interacted with ANLN, ARHGDIA, RTKN, ROCK2, and ARHGAP1 (Fig. 5A-F). Analysis of the relationship with the PI3K/AKT/mTOR signaling pathway based on the protein-protein interaction network showed that all of these genes are related, as shown in Fig. 5G.

## Discussion

Proteomic analysis has recently acquired popularity as a fundamental research method for identifying molecular markers for cancer detection. Furthermore, identifying and comparing cancer-related proteins is required for therapeutic development [20]. These findings lay the groundwork for future research into medication effectiveness and the processes of tumor tissue in xenograft mice models. PEC dramatically decreased cell proliferation and triggered cell cycle arrest, autophagy, and death in both the AGS and MKN28 human GC cell lines via the PI3K/AKT/mTOR pathway [15]. Furthermore, we carried out comparative proteomic analysis and identification at the cellular level, identified many genes and pathways in both AGS and MKN28 cells. In both cell lines, we discovered two identical genes, DDX4 and LRSAM1, as well as two additional PI3K pathway-related genes, PK3CB, and CIP2A [16].

We established a mouse model in which AGS cells were xenografted into BALB/c nude mice. PEC therapy decreased the tumor size and weight in a dose-dependent manner in vivo. Furthermore, the hematological and biochemical study findings for aspartate aminotransferase, alanine aminotransferase, urea, creatinine, triglycerides, and total cholesterol did not depart from normal values [21, 22]. This suggests that PEC is not harmful to non-cancerous cells.

We used 1-DE and coupled it to LC–MS/MS analysis to look at changes in protein levels in tumor tissue. LC–MS/MS analysis successfully identified 512 differentially expressed proteins. In response to PEC therapy, many key pathways surfaced in the AGS-xenograft mouse model, including PI3K pathway (hsa04150), apoptosis (hsa04210), mTOR signaling pathway (hsa04151), and cell cycle (P00013); this is consistent with our prior results. The GeneCodis database also verified several pathways associated to cancer molecular regulation, such as ferroptosis (hsa04216), aminoacyl-tRNA biosynthesis (hsa00970), necroptosis (hsa04217), Wnt signaling pathway (hsa04310 and P00057), and HIF-1 signaling pathway (hsa04150).

GYS1, HSP90AB1, HSP90B1, LAMB1, HSP90AA1, MAPK1, GNB1, LAMC1, PPP2R1A, PPP2R2A, RPS6, COL1A1, LAMA1, and EIF4E were associated with the PI3K-Akt signaling pathway, while RHOA, MAPK1, SLC3A2, RPS6, EIF4E, and CAMK2D were associated with the mTOR signaling pathway. In our current study, the PI3K/AKT/mTOR pathway was found to be related to other signaling pathways such as aminoacyl-tRNA biosynthesis (hsa00970), Wnt signaling pathway (hsa04310 and P00057), HIF-1 signaling pathway (hsa04066), and integrin signaling pathway (P00034). Cell cycle arrest, invasion, and apoptosis may be induced by regulating the PI3K/AKT/mTOR pathway [23, 24].

In PANTHER pathway analysis, the CCKR pathway map (P06959) included seven proteins: ELAVL1, RHOA, MAPK1, GNB1, RPS6, EIF4E, and YES1. CCKR is a cholecystokinin receptor that has been linked to gastrin [25], a circulating hormone that maintains the stomach mucosa while also acting as a powerful cell growth factor in biological processes such as proliferation and tumor transformation. When gastrin or CCK binds to receptors, it activates several signaling pathways that send mitogenic signals to the nucleus and stimulate cell proliferation [25–27].

KEGG pathway analysis also revealed four proteins implicated in ferroptosis (hsa04216), CP, VDAC2, PCBP1, and SLC3A2. Ferroptosis is a type of planned cell death that involves iron. This mechanism is distinguished by the accumulation of lipid peroxides, which is genetically and biochemically distinct from conventional cell death, such as apoptosis [28, 29]. These proteomic profiles of the tumor tissue from the AGS-xenograft mouse model injected with or without PEC may aid in the development of PEC-based treatment methods.

Prior to deciding on the six genes, we looked at the functions of 582 proteins from LC-MS/MS analysis in cancer and found that, similarly to earlier research, the PI3K/AKT/mTOR pathway and cancer cell death were linked. ANXA11, CAMK2D, CTSD, EIF4E, MAPK1, and RHOA were considered as potential genes. Annexin A11 (ANXA11) is a Ca2^+^-regulated phospholipid-binding protein that regulates cytokinesis, apoptosis, and exocytosis in malignancies [30]. Upregulation of ANXA11 causes ovarian and colorectal cancer development, recurrence, and treatment resistance [30]. Through the PDGFR and MAPK/p53 pathways, ANXA11 is implicated in cancer metastasis, invasion, and treatment resistance [31]. However, the mechanism of the ANXA11 has not been explicitly documented in GC.

The calcium signal controls a variety of cellular activities implicated in cancer growth, including proliferation and invasion [32]. The calcium signal is important in cell proliferation in the early G1 phase, which is mediated by Ca2^+^ activation of FOS, JUN, and MYC [32, 33]. The calcium/calmodulin-dependent kinase 2 delta (CAMK2D) is required for Ca2^+^ signals transduction in human cells [34]. CAMK2D regulates differentiation, proliferation, and apoptosis in cancer cells. According to current study findings, CAMK2D promotes GC cell formation and metastasis by generating metalloproteinase-9 (MMP-9) via the NF-kB and AKT pathways [34–36].

Cathepsin D (CTSD) is a lysosomal aspartyl protease that is ubiquitously distributed in lysosomes [37, 38]. The CTSD is transcribed from many locations, including an estrogen-regulated transcript. As a result, CTSD research in breast cancer has been active, confirming that this protein is connected to the mTOR pathway [37]. CTSD activates the metastasis and invasion-mediated activation of early growth response protein 1 and human telomerase reverse transcriptase (hTERT) in GC [39].

In cancer cells, the eukaryotic translation initiation factor 4E (EIF4E) controlled translation via PI3K/AKT/mTOR and Ras/MAPK/Mnk signaling pathways [40]. Translational control, which governs cell proliferation, death, and differentiation via upstream regulatory pathways such as PI3K and MAPK [40, 41], is critical for gene expression and cellular activities. PEC inhibited EIF4E activation in AGS and MKN28 human GC cell lines via the PI3K/AKT/mTOR pathway in a prior work [15].

The MAP kinase family induces the mitogen-activated protein kinase 1 (MAPK1), also known as ERK2 [42]. The ERK/MAPK pathway promotes cell proliferation and metastasis by increasing metalloproteinases (MMPs) [42]. Many investigations have found a link between anti-cancer drug effectiveness and ERK1/2 activation inhibition [43, 44].

The transforming protein RhoA (RHOA), also known as Ras homolog family member A, has been implicated in a variety of GC signaling pathways [45]. ST3GAL4 is expressed during GC cell movement, resulting in Sialyl-Lewis X production, RHOA activation, and c-Met on cell surfaces [45, 46]. RHOA, together with Rac and Cdc42, is activated in GC via the mTOR signaling pathway to stimulate invasion mediated by CXCL12, a ligand of CXCR4 [45, 47].

Therefore, PEC PEC is believed to the therapeutic potential for gastric cancer and regulates tumors via pathways such as calcium, MAPK, PI3K/AKT/mTOR, and others, based on LC–MS/MS analysis, GO analysis, and reconfirmation of the expression of 6 genes in cells of AGS-xenograft tumors.

## Conclusions

A broad proteome study of tumor tissues from the AGS-xenograft mouse model was undertaken. Using bioinformatics methods such as GO and protein-protein interaction, we studied the function and role of each protein. In animal studies, there was no difference in body weight or hematological alterations between the PEC test group and the control group. This might imply that PEC is not toxic in the body and will have less adverse effects during anticancer activity. Combining the results of LC–MS/MS and GO analysis, PEC controlled the expression of 582 proteins in the AGS-xenografted tumor, and it was shown to be implicated in pathways such as PI3K/AKT/mTOR, HIF-1, and Wnt, as well as the cell cycle, apoptosis, and ferroptosis. In the future, in-depth study on each route will be undertaken in order to identify a PEC-targeted pathway. As a result, our findings in this study provide a solid foundation for future clinical research into the effects of PEC on cancer.

## Supplementary Information

Below is the link to the electronic supplementary material.

Supplementary material 1 (PDF 447.1 kb)

## Data Availability

The datasets used and analyzed during the current study are available from the corresponding author on reasonable request.
