# Supplementary material for: Pectolinarigenin regulates the tumor-associated proteins in AGS-xenograft BALB/c nude mice
Source: Mol Biol Rep. 2024 Feb 16;51(1):305. doi: 10.1007/s11033-023-09046-4 (PMC10869406; doi:10.1007/s11033-023-09046-4)
Supplement: Supplementary file 1 — Supplementary material 1 (PDF 447.1 kb) [file 11033_2023_9046_MOESM1_ESM.pdf]

No.	Accesion No.	Description	Up/Down	Sum PEP Score	Coverage [%]	# Peptides	MW [kDa]	calc. pI	Abundances (Grouped): VC	Abundances (Grouped): PC	Abundances (Grouped): T1	Abundances (Grouped): T2
1	O43707	Alpha-actinin-4 [OS=Homo sapiens]	↓	534.462	65	48	104.8	5.44	134.5	97.3	90.8	77.4
2	P14625	Endoplasmin [OS=Homo sapiens]	↓	473.11	57	47	92.4	4.84	158.4	85.9	89	66.7
3	P14618-2	Isoform M1 of Pyruvate kinase PKM [OS=Homo sapiens]	↓	463.526	62	29	58	7.71	135.6	80	93.8	90.6
4	P60709	Actin, cytoplasmic 1 [OS=Homo sapiens]	↓	433.137	77	20	41.7	5.48	126.7	97	95.1	81.2
5	P12814-1	Alpha-actinin-1 [OS=Homo sapiens]	↓	409.082	64	42	103	5.41	127.2	100.5	96.2	76.2
6	Q00610-1	Clathrin heavy chain 1 [OS=Homo sapiens]	↓	362.667	40	47	191.5	5.69	110.5	114.1	107.6	67.8
7	P07900	Heat shock protein HSP 90-alpha [OS=Homo sapiens]	↓	343.475	52	37	84.6	5.02	147.1	82.1	95.6	75.1
8	P55072	Transitional endoplasmic reticulum ATPase [OS=Homo sapiens]	↓	342.344	59	33	89.3	5.26	135	104.4	96.2	64.5
9	P68371	Tubulin beta-4B chain [OS=Homo sapiens]	↓	316.999	73	24	49.8	4.89	146.1	90.7	87.5	75.7
10	P00558	phosphoglycerate kinase 1 [OS=Homo sapiens]	↑	309.017	69	24	44.6	8.1	74.3	121.3	85.3	119.2
11	P10809	60 kDa heat shock protein, mitochondrial [OS=Homo sapiens]	↓	305.116	58	28	61	5.87	139.6	84.3	98.5	77.6
12	P08238	Heat shock protein HSP 90-beta [OS=Homo sapiens]	↓	303.807	56	36	83.2	5.03	148	90.4	93.9	67.6
13	P22314	Ubiquitin-like modifier-activating enzyme 1 [OS=Homo sapiens]	↓	299.815	45	30	117.8	5.76	154.9	92.5	88.3	64.3
14	P68363	Tubulin alpha-1B chain [OS=Homo sapiens]	↓	258.546	64	20	50.1	5.06	139.2	90.3	91.2	79.3
15	P30740	Leukocyte elastase inhibitor [OS=Homo sapiens]	↓	241.624	57	20	42.7	6.28	118.5	78	112.7	90.8
16	Q01082-1	Spectrin beta chain, non-erythrocytic 1 [OS=Homo sapiens]	↓	239.824	19	34	274.4	5.57	132.6	91.3	98.3	77.9
17	Q13885	Tubulin beta-2A chain [OS=Homo sapiens]	↓	238.561	55	19	49.9	4.89	120.9	107.6	98.9	72.6
18	Q9BQE3	Tubulin alpha-1C chain [OS=Homo sapiens]	↓	238.347	64	20	49.9	5.1	136.4	100.2	87.9	75.5
19	P12956	X-ray repair cross-complementing protein 6 [OS=Homo sapiens]	↓	234.006	46	24	69.8	6.64	149.9	81.3	98	70.7
20	P00338-1	L-lactate dehydrogenase A chain [OS=Homo sapiens]	↑	204.572	73	21	36.7	8.27	76.8	118.8	89.1	115.4
21	P21333	Filamin-A [OS=Homo sapiens]	↓	204.041	20	34	280.6	6.06	123.8	118.5	85.2	72.6
22	Q15084-1	Protein disulfide-isomerase A6 [OS=Homo sapiens]	↓	198.108	43	15	48.1	5.08	151.5	89.5	86.9	72.1
23	P49411	elongation factor Tu, mitochondrial [OS=Homo sapiens]	↓	196.835	48	19	49.5	7.61	158.6	77.5	91.9	72.1
24	P61978	Heterogeneous nuclear ribonucleoprotein K [OS=Homo sapiens]	↓	186.74	52	19	50.9	5.54	137.3	91	90.2	81.5
25	P13010	X-ray repair cross-complementing protein 5 [OS=Homo sapiens]	↓	184.96	44	22	82.7	5.81	132.1	94.1	97.8	76
26	O75369-1	Filamin-B [OS=Homo sapiens]	↓	181.88	17	32	278	5.73	140.3	99.4	89.8	70.5
27	P68104	Elongation factor 1-alpha 1 [OS=Homo sapiens]	↓	180.323	53	16	50.1	9.01	124.1	102.1	97.7	76.1
28	P07098	Gastric triacylglycerol lipase [OS=Homo sapiens]	↓	179.373	53	13	45.2	7.33	121.9	84.1	127.5	66.5
29	P09429	High mobility group protein B1 [OS=Homo sapiens]	↑	178.422	44	10	24.9	5.74	75.3	98.1	102.5	124.1
30	P42704	Leucine-rich PPR motif-containing protein, mitochondrial [OS=Homo sa	↓	176.963	30	30	157.8	6.13	151.7	74.8	97.6	75.9
31	P11047	Laminin subunit gamma-1 [OS=Homo sapiens]	↑	169.249	16	22	177.5	5.12	29	204.7	36.2	130.1
32	Q9Y4L1	Hypoxia up-regulated protein 1 [OS=Homo sapiens]	↓	166.85	29	19	111.3	5.22	147.9	77.7	100.2	74.2
33	Q13263	Transcription intermediary factor 1-beta [OS=Homo sapiens]	↓	164.121	35	20	88.5	5.77	131.5	90.3	92.1	86.1
34	P51991-1	Heterogeneous nuclear ribonucleoprotein A3 [OS=Homo sapiens]	↑	158.043	39	17	39.6	9.01	87.7	135.4	87	89.9
35	Q13509	tubulin beta-3 chain [OS=Homo sapiens]	↓	155.987	38	14	50.4	4.93	119.3	93.3	108.9	78.5
36	Q99873	protein arginine N-methyltransferase 1 [OS=Homo sapiens]	↑	154.893	46	17	41.5	5.43	87.1	92.6	99.1	121.2
37	Q9Y490	Talin-1 [OS=Homo sapiens]	↓	153.017	14	21	269.6	6.07	124.7	104.8	105.4	65.1
38	P36578	60S ribosomal protein L4 [OS=Homo sapiens]	↑	148.526	40	17	47.7	11.06	16.6	104.2	131.9	147.4
39	Q96QK1	Vacuolar protein sorting-associated protein 35 [OS=Homo sapiens]	↓	145.882	28	17	91.6	5.49	151.4	79.5	91.1	78
40	Q7Z406-1	myosin-14 [OS=Homo sapiens]	↓	145.357	14	20	227.7	5.6	131.8	89.9	95.7	82.6
41	P14866	Heterogeneous nuclear ribonucleoprotein L [OS=Homo sapiens]	↓	141.058	41	14	64.1	8.22	139.5	96.4	87.6	76.5
42	Q07065	Cytoskeleton-associated protein 4 [OS=Homo sapiens]	↓	139.759	33	15	66	5.92	129.6	105.6	87.8	77
43	Q7KZF4	staphylococcal nuclease domain-containing protein 1 [OS=Homo sapier	↓	136.53	29	19	101.9	7.17	112.5	103.3	103.6	80.6
44	P36871-1	Phosphoglucomutase-1 [OS=Homo sapiens]	↓	133.924	50	19	61.4	6.76	138.4	100.4	92.3	68.9
45	P67809	Nuclease-sensitive element-binding protein 1 [OS=Homo sapiens]	↑	131.425	50	10	35.9	9.88	68.9	105.6	108	117.5
46	P07942	Laminin subunit beta-1 [OS=Homo sapiens]	↑	131.013	15	22	197.9	4.94	33.7	194.9	43.1	128.2
47	P62424	60S ribosomal protein L7a [OS=Homo sapiens]	↑	127.357	50	16	30	10.61	25.7	115.4	137.9	121
48	Q92841	Probable ATP-dependent RNA helicase DDX17 [OS=Homo sapiens]	↓	127.148	38	22	80.2	8.27	150.7	89.1	91.7	68.5
49	P62805	histone H4 [OS=Homo sapiens]	↑	125.5	53	9	11.4	11.36	33.6	113.3	111.7	141.4
50	O00299	chloride intracellular channel protein 1 [OS=Homo sapiens]	↓	125.427	78	14	26.9	5.17	135.7	83.7	99.2	81.4
51	P40939	Trifunctional enzyme subunit alpha, mitochondrial [OS=Homo sapiens]	↓	124.14	34	16	82.9	9.04	128.6	83.3	106.3	81.9
52	P00505	Aspartate aminotransferase, mitochondrial [OS=Homo sapiens]	↑	120.919	48	17	47.5	9.01	77.5	92.5	119.3	110.7
53	P13929-1	Beta-enolase [OS=Homo sapiens]	↓	120.231	45	12	47	7.71	102.3	80.6	175.4	41.6
54	P12882	Myosin-1 [OS=Homo sapiens]	↓	119.806	13	20	223	5.74	76.9	18.3	288.1	16.7
55	P07814	Bifunctional glutamate/proline--tRNA ligase [OS=Homo sapiens]	↓	118.906	21	22	170.5	7.33	133.2	88.1	105.7	73.1
56	Q01105-2	Isoform 2 of Protein SET [OS=Homo sapiens]	↑	118.705	39	8	32.1	4.22	75.4	96.8	107.3	120.5
57	P61158	actin-related protein 3 [OS=Homo sapiens]	↓	116.357	55	14	47.3	5.88	147.2	94.1	84	74.7
58	Q9UL46	proteasome activator complex subunit 2 [OS=Homo sapiens]	↓	114.311	68	12	27.4	5.73	127.6	91.4	100.9	80.1
59	P30153	serine/threonine-protein phosphatase 2A 65 kDa regulatory subunit A a	↓	113.911	35	15	65.3	5.11	131.4	87.8	99.3	81.5
60	P62158	Calmodulin [OS=Homo sapiens]	↑	111.686	77	10	16.8	4.22	71.5	95.8	99.2	133.4
61	P09874	Poly [ADP-ribose] polymerase 1 [OS=Homo sapiens]	↓	108.865	24	16	113	8.88	126.1	82.1	108.3	83.4
62	P54652	Heat shock-related 70 kDa protein 2 [OS=Homo sapiens]	↓	108.366	16	9	70	5.74	126.4	96.9	79.4	97.2

63	P67936	Tropomyosin alpha-4 chain [OS=Homo sapiens]	↑	106.018	42	13	28.5	4.69	78.3	90.9	95.8	135
64	Q15365	Poly(RC)-binding protein 1 [OS=Homo sapiens]	↓	105.802	63	13	37.5	7.09	139.4	89	93	78.7
65	P07910-1	Heterogeneous nuclear ribonucleoproteins C1/C2 [OS=Homo sapiens]	↑	105.271	39	12	33.7	5.08	76.1	113.1	79.5	131.3
66	P21796	voltage-dependent anion-selective channel protein 1 [OS=Homo sapien	↓	104.949	47	11	30.8	8.54	150.3	85.4	88.5	75.9
67	Q08211	Atp-dependent rna helicase a [OS=Homo sapiens]	↓	104.814	18	17	140.9	6.84	125.3	90.4	111	73.3
68	Q99623	Prohibitin-2 [OS=Homo sapiens]	↓	104.78	50	12	33.3	9.83	123.8	91	100.2	85
69	O75367-1	Core histone macro-H2A.1 [OS=Homo sapiens]	↑	104.583	48	12	39.6	9.79	70.7	101.7	116.4	111.2
70	Q02878	60S ribosomal protein L6 [OS=Homo sapiens]	↑	104.156	38	13	32.7	10.58	12.8	105.3	123.3	158.6
71	P26599	Polypyrimidine tract-binding protein 1 [OS=Homo sapiens]	↓	103.879	31	11	57.2	9.17	139.8	93.4	89.9	76.9
72	Q12906-1	Interleukin enhancer-binding factor 3 [OS=Homo sapiens]	↓	103.633	24	18	95.3	8.76	140.9	83.6	99.7	75.8
73	Q14980-1	nuclear mitotic apparatus protein 1 [OS=Homo sapiens]	↓	102.059	12	17	238.1	5.78	131.4	88.7	98.7	81.2
74	O15144	Actin-related protein 2/3 complex subunit 2 [OS=Homo sapiens]	↓	101.053	53	10	34.3	7.36	122.5	108.4	92.4	76.7
75	P35232	Prohibitin [OS=Homo sapiens]	↓	100.507	61	12	29.8	5.76	153.8	75.8	92.5	78
76	Q13200	26S proteasome non-ATPase regulatory subunit 2 [OS=Homo sapiens]	↓	98.233	24	15	100.1	5.2	129.9	116.7	87.9	65.6
77	P62136-1	serine/threonine-protein phosphatase PP1-alpha catalytic subunit [OS=H	↓	97.692	50	13	37.5	6.33	116.6	113.6	94.2	75.6
78	P51659-1	peroxisomal multifunctional enzyme type 2 [OS=Homo sapiens]	↓	96.358	32	15	79.6	8.84	134.2	89.8	96.1	79.9
79	O60701	UDP-glucose 6-dehydrogenase [OS=Homo sapiens]	↓	95.365	37	12	55	7.12	160.5	70	103.5	66
80	Q71DI3	histone H3.2 [OS=Homo sapiens]	↑	95.07	43	5	15.4	11.27	45.8	124.3	111.6	118.3
81	P18124	60S ribosomal protein L7 [OS=Homo sapiens]	↑	94.313	51	13	29.2	10.65	18.2	110.4	126.2	145.2
82	P06753	Tropomyosin alpha-3 chain [OS=Homo sapiens]	↑	92.67	27	12	32.9	4.72	63.8	111.1	136.5	88.7
83	Q00341-1	Vigilin [OS=Homo sapiens]	↓	92.093	17	15	141.4	6.87	131.2	89.3	98	81.5
84	Q9BS26	Endoplasmic reticulum resident protein 44 [OS=Homo sapiens]	↓	91.911	37	10	46.9	5.26	152.1	89.7	94.9	63.3
85	P62906	60S ribosomal protein L10A [OS=Homo sapiens]	↑	91.226	38	8	24.8	9.94	23.5	116.2	124.8	135.5
86	P68431	Histone H3.1 [OS=Homo sapiens]	↑	91.046	43	5	15.4	11.12	50.1	111.9	113.7	124.2
87	P08670	Vimentin [OS=Homo sapiens]	↑	90.928	35	15	53.6	5.12	57.2	166.2	80.1	96.5
88	P07339	Cathepsin D [OS=Homo sapiens]	↓	90.776	34	12	44.5	6.54	138.2	101.9	87.9	72
89	Q15181	Inorganic pyrophosphatase [OS=Homo sapiens]	↓	90.574	55	11	32.6	5.86	149	91.5	92.5	67
90	P40227-1	T-complex protein 1 subunit zeta [OS=Homo sapiens]	↓	88.452	32	11	58	6.68	142.5	94	92.9	70.6
91	Q14974	Importin subunit beta-1 [OS=Homo sapiens]	↓	88.278	20	12	97.1	4.78	136	95.4	88.5	80.2
92	P62140	Serine/threonine-protein phosphatase PP1-beta catalytic subunit [OS=H	↓	86.762	43	11	37.2	6.19	129	105.6	97.8	67.5
93	P43243	Matrin-3 [OS=Homo sapiens]	↓	86.447	21	12	94.6	6.25	136.4	99	87.9	76.7
94	P09488	Glutathione S-transferase Mu 1 [OS=Homo sapiens]	↑	86.013	51	11	25.7	6.7	81.6	131	86.5	100.9
95	Q12905	Interleukin enhancer-binding factor 2 [OS=Homo sapiens]	↓	85.985	46	11	43	5.26	150.1	80.3	97.1	72.5
96	P18206	Vinculin [OS=Homo sapiens]	↓	84.518	18	14	123.7	5.66	125.8	107.5	91.6	75.1
97	Q1KMD3	heterogeneous nuclear ribonucleoprotein U-like protein 2 [OS=Homo sa	↓	82.744	20	11	85.1	4.91	123.8	89.5	109.7	77
98	P16402	Histone H1.3 [OS=Homo sapiens]	↑	82.632	24	8	22.3	11.02	24	116.1	111.6	148.3
99	P48643	T-complex protein 1 subunit epsilon [OS=Homo sapiens]	↓	81.993	35	13	59.6	5.66	155.7	86.7	86.8	70.8
100	P26583	High mobility group protein B2 [OS=Homo sapiens]	↑	81.916	37	9	24	7.81	90.1	135.9	84.3	89.6
101	P09493-10	Isoform 10 of Tropomyosin alpha-1 chain [OS=Homo sapiens]	↑	80.821	30	12	32.8	4.77	81.6	101.7	128	88.7
102	Q01518-1	adenylyl cyclase-associated protein 1 [OS=Homo sapiens]	↓	80.441	36	12	51.9	8.06	136.1	91.9	98.9	73.1
103	Q9NSE4	Isoleucine--tRNA ligase, mitochondrial [OS=Homo sapiens]	↓	80.209	26	17	113.7	7.2	153.3	84.1	99.1	63.5
104	P05388	60S acidic ribosomal protein P0 [OS=Homo sapiens]	↓	80.031	45	10	34.3	5.97	132.4	99.6	96.6	71.4
105	P49368-1	T-complex protein 1 subunit gamma [OS=Homo sapiens]	↓	79.864	30	13	60.5	6.49	148	89	93.7	69.3
106	P78371-1	T-complex protein 1 subunit beta [OS=Homo sapiens]	↓	79.003	26	9	57.5	6.46	154.3	82.7	95.9	67.1
107	P08779	Keratin, type I cytoskeletal 16 [OS=Homo sapiens]	↓	77.167	33	13	51.2	5.05	119.7	85	141.6	53.7
108	P47756-2	Isoform 2 of F-actin-capping protein subunit beta [OS=Homo sapiens]	↓	75.009	35	8	30.6	6	123.7	93.3	99.8	83.2
109	O00571	ATP-dependent RNA helicase DDX3X [OS=Homo sapiens]	↓	74.853	24	12	73.2	7.18	125.3	100.6	100.2	74
110	Q16777	Histone H2A type 2-C [OS=Homo sapiens]	↑	74.593	58	5	14	10.9	65	105	98.8	131.2
111	P52565-1	rho GDP-dissociation inhibitor 1 [OS=Homo sapiens]	↓	74.556	43	7	23.2	5.11	134.2	103.3	88	74.5
112	P53621-1	coatomer subunit alpha [OS=Homo sapiens]	↓	73.791	17	16	138.3	7.66	135.7	90.4	97.1	76.9
113	P27816-1	Microtubule-associated protein 4 [OS=Homo sapiens]	↑	73.765	11	9	120.9	5.43	68.7	102.4	101.8	127.1
114	P08758	annexin A5 [OS=Homo sapiens]	↑	73.395	34	11	35.9	5.05	62	169.5	80.3	88.3
115	Q9Y3Z3	deoxynucleoside triphosphate triphosphohydrolase SAMHD1 [OS=Homo	↓	73.254	30	12	72.2	7.14	160.3	83.9	94.4	61.4
116	P25391	Laminin subunit alpha-1 [OS=Homo sapiens]	↑	72.967	5	13	336.9	6.35	27.8	177.4	49.7	145.1
117	P52907	F-actin-capping protein subunit alpha-1 [OS=Homo sapiens]	↓	72.714	46	9	32.9	5.69	126.3	84.5	104.7	84.5
118	Q92499	ATP-dependent RNA helicase DDX1 [OS=Homo sapiens]	↓	72.673	20	10	82.4	7.23	162.6	78.9	89	69.5
119	P46777	60S ribosomal protein L5 [OS=Homo sapiens]	↑	72.158	33	8	34.3	9.72	60.5	117.2	118.3	104
120	Q9UBT2	SUMO-activating enzyme subunit 2 [OS=Homo sapiens]	↓	70.63	30	11	71.2	5.29	152.8	76.6	97.3	73.3
121	P26440	Isovaleryl-CoA dehydrogenase, mitochondrial [OS=Homo sapiens]	↑	70.593	34	10	46.3	8.19	74.5	92.6	111.2	121.7
122	P06732	Creatine kinase M-type [OS=Homo sapiens]	↓	70.341	30	8	43.1	7.25	97.4	26.6	255	21.1
123	P39656	Dolichyl-diphosphooligosaccharide--protein glycosyltransferase 48 kDa s	↓	70.216	45	13	50.8	6.55	120.5	92.5	104.9	82.1
124	P22695	Cytochrome b-c1 complex subunit 2, mitochondrial [OS=Homo sapiens]	↓	69.685	36	8	48.4	8.63	141.8	79.5	108.1	70.6
125	O95865	N(G),N(G)-dimethylarginine dimethylaminohydrolase 2 [OS=Homo sapie	↓	69.434	75	11	29.6	6.01	134.2	92.6	87	86.2

126	P23229-1	integrin alpha-6 [OS=Homo sapiens]	↓	69.311	14	12	126.5	6.61	129.9	94.7	98.8	76.7
127	O14579-1	coatomer subunit epsilon [OS=Homo sapiens]	↓	68.946	38	6	34.5	5.12	129.5	96.2	92.4	81.9
128	P31930	Cytochrome b-c1 complex subunit 1, mitochondrial [OS=Homo sapiens]	↓	68.403	33	10	52.6	6.37	158.6	72.9	95.9	72.7
129	P40121	Macrophage-capping protein [OS=Homo sapiens]	↓	68.053	32	7	38.5	6.19	132.9	91.9	93.2	82
130	P55084	Trifunctional enzyme subunit beta, mitochondrial [OS=Homo sapiens]	↓	66.823	43	14	51.3	9.41	139.3	85.3	99.6	75.7
131	Q9NYU2-1	UDP-glucose:glycoprotein glucosyltransferase 1 [OS=Homo sapiens]	↓	66.371	12	12	177.1	5.63	151.4	79.9	108.9	59.7
132	P28331-1	NADH-ubiquinone oxidoreductase 75 kDa subunit, mitochondrial [OS=H	↓	65.916	20	9	79.4	6.23	150.5	87	96.6	65.9
133	P07384	Calpain-1 catalytic subunit [OS=Homo sapiens]	↓	65.262	20	12	81.8	5.67	114.9	102.6	102.3	80.2
134	Q15233	Non-POU domain-containing octamer-binding protein [OS=Homo sapie	↓	65.147	19	6	54.2	8.95	128.4	99.7	101.8	70.2
135	Q15019	septin-2 [OS=Homo sapiens]	↓	64.822	32	7	41.5	6.6	135.9	113.1	86	64.9
136	P09104	Gamma-enolase [OS=Homo sapiens]	↑	64.817	20	6	47.2	5.03	72.1	157.3	59.4	111.1
137	P43686	26S proteasome regulatory subunit 6B [OS=Homo sapiens]	↓	64.455	33	7	47.3	5.21	162.6	81.5	90.6	65.2
138	P17931	Galectin-3 [OS=Homo sapiens]	↓	64.343	28	6	26.1	8.56	125.3	106.8	82.9	85
139	Q9Y265	RuvB-like 1 [OS=Homo sapiens]	↓	62.453	28	9	50.2	6.42	143.6	93.4	90.6	72.4
140	P39023	60S ribosomal protein L3 [OS=Homo sapiens]	↑	62.388	28	8	46.1	10.18	30.4	111.7	131.1	126.7
141	Q9UKV3-1	Apoptotic chromatin condensation inducer in the nucleus [OS=Homo sa	↓	62.276	9	10	151.8	6.43	119.5	72.2	102.8	105.6
142	P62826	GTP-binding nuclear protein RAN [OS=Homo sapiens]	↓	61.387	39	7	24.4	7.49	120.8	97.3	103	78.8
143	P36776	Lon protease homolog, mitochondrial [OS=Homo sapiens]	↓	61.352	16	9	106.4	6.39	139.6	85.1	102.4	72.9
144	P62241	40S ribosomal protein S8 [OS=Homo sapiens]	↑	61.294	38	7	24.2	10.32	44	126.3	126.5	103.2
145	P09493-5	Isoform 5 of Tropomyosin alpha-1 chain [OS=Homo sapiens]	↑	61.084	27	9	28.4	4.77	32.6	119.7	107.5	140.3
146	Q86VP6-1	cullin-associated nedd8-dissociated protein 1 [OS=Homo sapiens]	↓	60.399	13	10	136.3	5.78	119.5	104.8	98	77.7
147	Q03013-1	glutathione S-transferase mu 4 [OS=Homo sapiens]	↑	60.171	36	7	25.5	5.9	62.6	105.9	111.6	119.9
148	Q15149-1	plectin [OS=Homo sapiens]	↓	60.097	3	9	531.5	5.96	134.6	95	95.1	75.4
149	P45974-1	Ubiquitin carboxyl-terminal hydrolase 5 [OS=Homo sapiens]	↓	60.02	15	9	95.7	5.03	157	85.5	90.7	66.8
150	P21964-1	Catechol O-methyltransferase [OS=Homo sapiens]	↓	59.996	39	7	30	5.47	123.8	96	102.5	77.7
151	P11413-1	Glucose-6-phosphate 1-dehydrogenase [OS=Homo sapiens]	↓	59.911	26	12	59.2	6.84	127.3	87.1	105.9	79.7
152	Q96HE7	ERO1-like protein alpha [OS=Homo sapiens]	↓	59.35	30	10	54.4	5.68	135.7	109.1	76.2	79
153	Q15029	116 kDa U5 small nuclear ribonucleoprotein component [OS=Homo sap	↓	58.96	14	10	109.4	5	132.9	101.7	93.6	71.8
154	P84243	histone H3.3 [OS=Homo sapiens]	↑	58.748	43	5	15.3	11.27	55.1	114.6	108.9	121.4
155	P16989-1	Y-box-binding protein 3 [OS=Homo sapiens]	↑	58.506	16	5	40.1	9.77	73.9	114.1	96.2	115.8
156	Q9Y678	Coatomer subunit gamma-1 [OS=Homo sapiens]	↓	58.494	19	12	97.7	5.47	124.7	94.5	102.3	78.5
157	Q86V81	THO complex subunit 4 [OS=Homo sapiens]	↑	57.414	31	4	26.9	11.15	49.6	104.6	114.1	131.6
158	P46782	40S ribosomal protein S5 [OS=Homo sapiens]	↓	56.426	42	6	22.9	9.72	122.1	108.8	92.6	76.5
159	P13533	myosin-6 [OS=Homo sapiens]	↑	56.386	6	10	223.6	5.73	38.6	237	124.4	
160	Q13087	Protein disulfide-isomerase A2 [OS=Homo sapiens]	↓	56.368	21	8	58.2	4.98	202.2	66.1	74.4	57.3
161	Q9Y281-1	Cofilin-2 [OS=Homo sapiens]	↑	56.189	31	5	18.7	7.88	73.4	168	82.7	75.9
162	Q15366-1	Poly(rC)-binding protein 2 [OS=Homo sapiens]	↓	54.909	25	7	38.6	6.79	138.2	90	93.9	77.9
163	Q9UJZ1	Stomatin-like protein 2, mitochondrial [OS=Homo sapiens]	↓	54.893	43	9	38.5	7.39	142	82	87.9	88.1
164	Q02790	Peptidyl-prolyl cis-trans isomerase FKBP4 [OS=Homo sapiens]	↓	54.729	31	10	51.8	5.43	143.7	83	94.8	78.4
165	P45880	Voltage-dependent anion-selective channel protein 2 [OS=Homo sapien	↓	53.53	30	7	31.5	7.56	152.2	95.8	91.6	60.4
166	Q8WXF1	Paraspeckle component 1 [OS=Homo sapiens]	↓	53.296	14	6	58.7	6.67	131.1	90.3	97.8	80.9
167	P63010-1	AP-2 complex subunit beta [OS=Homo sapiens]	↓	52.998	12	8	104.5	5.38	138.3	93.3	96.5	71.9
168	Q06210-1	glutamine--fructose-6-phosphate aminotransferase [isomerizing] 1 [OS=	↓	52.865	19	9	78.8	7.11	127.8	97	98.2	77
169	P34896-1	Serine hydroxymethyltransferase, cytosolic [OS=Homo sapiens]	↑	52.75	24	7	53	7.71	66.4	88.1	134.5	111.1
170	P50995	annexin A11 [OS=Homo sapiens]	↓	52.05	21	9	54.4	7.65	138.3	84.6	96.1	81.1
171	O75489	NADH dehydrogenase [ubiquinone] iron-sulfur protein 3, mitochondrial	↓	51.948	31	7	30.2	7.5	157	77.2	89.7	76
172	P62314	Small nuclear ribonucleoprotein Sm D1 [OS=Homo sapiens]	↑	51.739	55	5	13.3	11.56	44.5	103.1	122.7	129.6
173	P01008	Antithrombin-III [OS=Homo sapiens]	↑	50.61	18	8	52.6	6.71	74.6	141.1	83.4	100.9
174	P62081	40S ribosomal protein S7 [OS=Homo sapiens]	↓	50.283	52	7	22.1	10.1	135.5	91.5	97.1	75.9
175	P56537-1	eukaryotic translation initiation factor 6 [OS=Homo sapiens]	↓	50.067	40	6	26.6	4.68	136.3	90.1	89.1	84.5
176	P11217	Glycogen phosphorylase, muscle form [OS=Homo sapiens]	↓	49.861	20	14	97	7.03	76.1	23.6	269.9	30.4
177	Q9Y230	RuvB-like 2 [OS=Homo sapiens]	↓	49.224	23	9	51.1	5.64	160.9	75.6	92	71.5
178	P02538	Keratin, type II cytoskeletal 6A [OS=Homo sapiens]	↓	48.793	17	10	60	8	93.9	58.7	183.8	63.6
179	Q9HC38	Glyoxalase domain-containing protein 4 [OS=Homo sapiens]	↓	48.622	39	11	34.8	5.6	144.4	90.9	88.6	76
180	Q14195-1	Dihydropyrimidinase-related protein 3 [OS=Homo sapiens]	↑	48.6	13	5	61.9	6.49	53.5	176.4	75.9	94.2
181	P38606	V-type proton ATPase catalytic subunit A [OS=Homo sapiens]	↓	48.443	16	8	68.3	5.52	124.4	100.6	97.1	77.8
182	O95336	6-phosphogluconolactonase [OS=Homo sapiens]	↓	48.282	55	7	27.5	6.05	163.4	77.6	81.5	77.6
183	P13797	Plastin-3 [OS=Homo sapiens]	↓	47.946	18	9	70.8	5.6	123.8	102.4	98.1	75.6
184	P01892	HLA class I histocompatibility antigen, A-2 alpha chain [OS=Homo sapie	↓	47.94	25	6	40.9	6.99	166.5	73	92.3	68.2
185	O60271	C-jun-amino-terminal kinase-interacting protein 4 [OS=Homo sapiens]	↓	47.775	10	9	146.1	5.15	143.2	69.5	101	86.3
186	P16401	Histone H1.5 [OS=Homo sapiens]	↑	47.701	22	6	22.6	10.92	16.1	108.8	113.7	161.4
187	P62191	26S proteasome regulatory subunit 4 [OS=Homo sapiens]	↓	47.501	19	6	49.2	6.21	136.1	83.7	102.6	77.7
188	P41250	Glycine--tRNA ligase [OS=Homo sapiens]	↓	47.31	13	7	83.1	7.03	116.9	112.8	104.8	65.6

189	O14744	Protein arginine N-methyltransferase 5 [OS=Homo sapiens]	↑	46.849	22	9	72.6	6.29	67	88	121.3	123.7
190	Q9NTK5-1	obg-like ATPase 1 [OS=Homo sapiens]	↓	46.837	19	6	44.7	7.81	155.1	79.2	93.4	72.3
191	P04179	Superoxide dismutase [Mn], mitochondrial [OS=Homo sapiens]	↓	46.484	27	6	24.7	8.25	129.1	99	90	81.8
192	P98179	RNA-binding protein 3 [OS=Homo sapiens]	↑	46.463	41	3	17.2	8.91	66	102	101.1	130.9
193	Q9UNM6	26S proteasome non-ATPase regulatory subunit 13 [OS=Homo sapiens]	↓	46.405	23	7	42.9	5.81	142.2	103.1	94	60.7
194	P21291	Cysteine and glycine-rich protein 1 [OS=Homo sapiens]	↑	46.298	42	6	20.6	8.57	75.9	119	98.4	106.7
195	P13796	Plastin-2 [OS=Homo sapiens]	↓	46.243	20	9	70.2	5.43	150.5	82.9	97.3	69.3
196	P18085	ADP-ribosylation factor 4 [OS=Homo sapiens]	↓	46.108	41	6	20.5	7.14	128.7	94.8	101.8	74.7
197	O95834	echinoderm microtubule-associated protein-like 2 [OS=Homo sapiens]	↓	45.257	13	6	70.6	6.32	105	84.4	116.5	94.1
198	P50914	60S ribosomal protein L14 [OS=Homo sapiens]	↑	45.018	27	5	23.4	10.93	49.3	107	116.2	127.5
199	P18754-1	Regulator of chromosome condensation [OS=Homo sapiens]	↓	44.927	15	5	44.9	7.52	127.6	91.3	109.3	71.7
200	Q8IUE6	Histone H2A type 2-B [OS=Homo sapiens]	↑	44.918	58	5	14	10.89	69.7	98.8	98.1	133.4
201	Q9Y3I0	tRNA-splicing ligase RtcB homolog [OS=Homo sapiens]	↓	44.786	20	6	55.2	7.23	147.8	79.9	88.3	83.9
202	Q13148-1	TAR DNA-binding protein 43 [OS=Homo sapiens]	↓	44.675	27	6	44.7	6.19	135.2	95.1	90.4	79.3
203	P09382	Galectin-1 [OS=Homo sapiens]	↑	44.592	64	6	14.7	5.5	85.9	137	81.6	95.5
204	P29966	Myristoylated alanine-rich C-kinase substrate [OS=Homo sapiens]	↓	43.738	30	4	31.5	4.45	117.4	73.9	97	111.7
205	Q9UMS4	Pre-mRNA-processing factor 19 [OS=Homo sapiens]	↓	42.578	18	7	55.1	6.61	125.6	89.1	110.9	74.4
206	P62195-1	26S proteasome regulatory subunit 8 [OS=Homo sapiens]	↓	41.605	24	7	45.6	7.55	136.6	84.5	103.4	75.5
207	Q14152	Eukaryotic translation initiation factor 3 subunit A [OS=Homo sapiens]	↓	41.193	9	9	166.5	6.79	149.1	76.8	105.4	68.7
208	Q13409-1	cytoplasmic dynein 1 intermediate chain 2 [OS=Homo sapiens]	↓	41.002	15	5	71.4	5.2	141.1	99.2	90.7	69
209	P17655-1	Calpain-2 catalytic subunit [OS=Homo sapiens]	↓	41	15	7	79.9	4.98	143.7	83.4	96.2	76.6
210	P54578	Ubiquitin carboxyl-terminal hydrolase 14 [OS=Homo sapiens]	↓	40.578	20	7	56	5.3	152.5	88.6	93.7	65.3
211	P61353	60S ribosomal protein L27 [OS=Homo sapiens]	↑	40.42	38	5	15.8	10.56	32.3	111.1	127.1	129.5
212	Q15459	splicing factor 3A subunit 1 [OS=Homo sapiens]	↓	40.331	15	9	88.8	5.22	145.7	83.1	92.3	78.9
213	P09661	U2 small nuclear ribonucleoprotein A' [OS=Homo sapiens]	↓	39.747	44	6	28.4	8.62	129.7	93.8	93.1	83.4
214	P0CG39	POTE ankyrin domain family member J [OS=Homo sapiens]	↓	39.694	8	5	117.3	5.97	126.2	102.7	90.5	80.6
215	Q16718	NADH dehydrogenase [ubiquinone] 1 alpha subcomplex subunit 5 [OS=	↓	39.49	39	4	13.5	5.99	137.5	82.8	101	78.7
216	O43252	bifunctional 3'-phosphoadenosine 5'-phosphosulfate synthase 1 [OS=Hc	↓	39.389	16	7	70.8	6.86	168.1	85.9	84.6	61.3
217	Q16658	Fascin [OS=Homo sapiens]	↑	39.125	19	5	54.5	7.24	56.1	153.4	77.3	113.2
218	P17980	26S proteasome regulatory subunit 6A [OS=Homo sapiens]	↓	39.116	27	7	49.2	5.24	132.7	94.5	96.9	75.9
219	P62917	60S ribosomal protein L8 [OS=Homo sapiens]	↑	39.095	27	7	28	11.03	28.8	108.2	120	143
220	Q9H3P7	Golgi resident protein GCP60 [OS=Homo sapiens]	↓	39.042	13	5	60.6	5.06	130.3	90.9	102.5	76.4
221	Q99832	T-complex protein 1 subunit eta [OS=Homo sapiens]	↓	38.902	18	7	59.3	7.65	122.4	91.8	97.4	88.3
222	Q96AB3	isochorismatase domain-containing protein 2 [OS=Homo sapiens]	↓	38.839	59	6	22.3	7.77	133.7	82.1	106.9	77.3
223	P13489	Ribonuclease inhibitor [OS=Homo sapiens]	↓	38.702	24	6	49.9	4.82	168	99.1	73.9	59
224	Q13126-1	S-methyl-5'-thioadenosine phosphorylase [OS=Homo sapiens]	↑	38.607	34	6	31.2	7.18	77.4	103.1	99.5	120
225	P14868	Aspartate--tRNA ligase, cytoplasmic [OS=Homo sapiens]	↓	37.951	20	8	57.1	6.55	130.1	87.7	101.6	80.6
226	Q14019	coactosin-like protein [OS=Homo sapiens]	↓	37.943	60	7	15.9	5.67	136.8	97.7	96.1	69.4
227	P02533	Keratin, type I cytoskeletal 14 [OS=Homo sapiens]	↓	37.79	17	8	51.5	5.16	99.7	86.4	136.1	77.7
228	P09496-2	Isoform Non-brain of Clathrin light chain A [OS=Homo sapiens]	↑	37.443	25	5	23.6	4.53	64.1	82.9	83.8	169.3
229	P51665	26S proteasome non-ATPase regulatory subunit 7 [OS=Homo sapiens]	↓	37.326	24	5	37	6.77	131.7	106	89.1	73.3
230	P18465	HLA class I histocompatibility antigen, B-57 alpha chain [OS=Homo sap	↓	37.063	18	5	40.2	6.3	141.6	86.5	97.3	74.6
231	Q9BR76	Coronin-1B [OS=Homo sapiens]	↓	37.004	26	7	54.2	5.88	146.7	99.7	89.2	64.3
232	Q9UKM9-1	RNA-binding protein Raly [OS=Homo sapiens]	↓	36.863	25	7	32.4	9.17	129.3	76.9	118.5	75.3
233	P42224-1	Signal transducer and activator of transcription 1-alpha/beta [OS=Homc	↓	36.8	11	7	87.3	6.05	141.3	99.6	93.1	66.1
234	Q8NC51-1	Plasminogen activator inhibitor 1 RNA-binding protein [OS=Homo sapie	↑	36.767	17	5	44.9	8.65	70.7	94.8	115.8	118.7
235	O94979	Protein transport protein Sec31A [OS=Homo sapiens]	↓	36.564	8	8	132.9	6.89	148.3	79.1	92.1	80.4
236	Q8WUM4	Programmed cell death 6-interacting protein [OS=Homo sapiens]	↓	36.4	13	8	96	6.52	155.6	87.3	84.1	73
237	P69905	Hemoglobin subunit alpha [OS=Homo sapiens]	↑	36.284	27	5	15.2	8.68	84.8	102.3	102.7	110.2
238	O00303	Eukaryotic translation initiation factor 3 subunit F [OS=Homo sapiens]	↓	35.945	14	3	37.5	5.45	159.1	84	86.6	70.4
239	P35613	Basigin [OS=Homo sapiens]	↓	35.882	17	5	42.2	5.66	134.3	73.7	104.1	87.8
240	P62750	60S ribosomal protein L23a [OS=Homo sapiens]	↑	35.834	34	6	17.7	10.45	56.1	116.3	113.1	114.5
241	P31146	Coronin-1A [OS=Homo sapiens]	↓	35.59	19	4	51	6.68	143.8	123.4	85.6	47.2
242	P14543-1	Nidogen-1 [OS=Homo sapiens]	↑	35.547	5	5	136.3	5.29	19.4	225.2	29.6	125.8
243	P61313-1	60S ribosomal protein L15 [OS=Homo sapiens]	↑	35.399	30	6	24.1	11.62	11.9	112.2	144.1	131.8
244	P32322	Pyrroline-5-carboxylate reductase 1, mitochondrial [OS=Homo sapiens]	↓	35.298	16	3	33.3	7.61	135.4	94.9	98.9	70.8
245	Q15102	Platelet-activating factor acetylhydrolase IB subunit gamma [OS=Homo	↓	35.276	36	5	25.7	6.84	145.9	87.6	90.5	76
246	P00492	Hypoxanthine-guanine phosphoribosyltransferase [OS=Homo sapiens]	↓	35.066	34	6	24.6	6.68	124.1	85.6	98.3	92
247	P61160-1	Actin-related protein 2 [OS=Homo sapiens]	↓	35.026	22	5	44.7	6.74	120.3	111.6	96.9	71.2
248	P54136-1	arginine--tRNA ligase, cytoplasmic [OS=Homo sapiens]	↓	34.974	13	7	75.3	6.68	134.9	88.1	97.2	79.9
249	Q9BWS9-1	Chitinase domain-containing protein 1 [OS=Homo sapiens]	↓	34.721	32	7	44.9	8.63	153	69	120.4	57.6
250	Q07020	60S ribosomal protein L18 [OS=Homo sapiens]	↑	34.658	35	7	21.6	11.72	15.7	122.5	119.2	142.6
251	P49588	Alanine--tRNA ligase, cytoplasmic [OS=Homo sapiens]	↓	34.639	8	6	106.7	5.53	187.7	68.2	83.5	60.5

252	P55735-1	protein SEC13 homolog [OS=Homo sapiens]	↓	34.602	25	5	35.5	5.48	116.9	101.1	104.9	77.1
253	Q5JWF2-1	Guanine nucleotide-binding protein G(S) subunit alpha isoforms XLas [C	↑	34.409	8	7	111	5.03	85.6	132.3	80.1	102
254	P61326	Protein mago nashi homolog [OS=Homo sapiens]	↓	34.378	47	5	17.2	6.11	128.3	114.1	100.1	57.5
255	O60749	Sorting nexin-2 [OS=Homo sapiens]	↓	34.107	12	5	58.4	5.12	124.4	103.5	95.2	76.9
256	O75643-1	U5 small nuclear ribonucleoprotein 200 kDa helicase [OS=Homo sapien	↓	34.07	4	6	244.4	6.06	140.4	88.7	89.9	81
257	Q07960	rho GTPase-activating protein 1 [OS=Homo sapiens]	↓	34.016	26	6	50.4	6.29	139.9	87.5	98.7	74
258	P62753	40S RIBOSOMAL PROTEIN S6 [OS=Homo sapiens]	↑	33.987	19	4	28.7	10.84	55.5	126	116.7	101.8
259	O15371	Eukaryotic translation initiation factor 3 subunit D [OS=Homo sapiens]	↑	33.736	15	6	63.9	6.05	55.5	159.4	70.5	114.6
260	O60664-1	Perilipin-3 [OS=Homo sapiens]	↓	33.661	25	5	47	5.44	118.4	63.4	108.3	109.8
261	P02675	Fibrinogen beta chain [OS=Homo sapiens]	↑	33.634	14	6	55.9	8.27	45.9	186.9	60	107.2
262	P62277	40S ribosomal protein S13 [OS=Homo sapiens]	↑	33.524	30	5	17.2	10.54	50.4	123.7	111.4	114.5
263	Q9BUT1-1	3-hydroxybutyrate dehydrogenase type 2 [OS=Homo sapiens]	↑	33.361	27	5	26.7	7.65	66.4	104.8	101.3	127.4
264	Q96FW1	Ubiquitin thioesterase otub1 [OS=Homo sapiens]	↓	33.346	27	6	31.3	4.94	153.6	93	85.8	67.7
265	Q16891	MICOS complex subunit Mic60 [OS=Homo sapiens]	↓	33.319	8	5	83.6	6.48	160.5	81.2	91	67.3
266	P07741-1	Adenine phosphoribosyltransferase [OS=Homo sapiens]	↓	33.243	37	5	19.6	6.02	162.7	80.4	89.4	67.4
267	P47897	glutamine--tRNA ligase [OS=Homo sapiens]	↓	33.218	12	7	87.7	7.15	180.2	78.7	88.9	52.1
268	Q12931	heat shock protein 75 kDa, mitochondrial [OS=Homo sapiens]	↓	33.194	10	5	80.1	8.21	157.8	72.9	96.2	73.1
269	Q96A32	Myosin regulatory light chain 2, skeletal muscle isoform [OS=Homo sap	↓	32.78	41	6	19	5.01	130.3	48.8	168.4	52.5
270	Q9UHX1-1	poly(U)-binding-splicing factor PUF60 [OS=Homo sapiens]	↓	32.777	13	6	59.8	5.29	133.1	89.5	99.8	77.7
271	P83731	60S ribosomal protein L24 [OS=Homo sapiens]	↑	32.027	31	5	17.8	11.25	53.9	126.6	107.7	111.8
272	P06730	Eukaryotic translation initiation factor 4E [OS=Homo sapiens]	↓	31.862	21	5	25.1	6.15	140.1	84.9	96.6	78.4
273	Q14257	Reticulocalbin-2 [OS=Homo sapiens]	↑	31.755	23	4	36.9	4.4	87.6	99.5	95.5	117.4
274	P62249	40S ribosomal protein S16 [OS=Homo sapiens]	↑	31.714	36	6	16.4	10.21	56.2	126.3	115.4	102.1
275	Q16851	UTP--glucose-1-phosphate uridylyltransferase [OS=Homo sapiens]	↓	31.416	18	7	56.9	8.15	139.6	94.7	92.9	72.7
276	P56192	Methionine--tRNA ligase, cytoplasmic [OS=Homo sapiens]	↓	31.118	8	6	101.1	6.16	150.9	83.1	94.4	71.6
277	P09543-1	2',3'-cyclic-nucleotide 3'-phosphodiesterase [OS=Homo sapiens]	↓	31.016	19	5	47.5	9.07	122.3	102.3	93	82.4
278	Q12959	Disks large homolog 1 [OS=Homo sapiens]	↓	30.908	10	5	100.4	5.76	111.2	101.6	87.3	99.9
279	Q99829	Copine-1 [OS=Homo sapiens]	↓	30.894	15	6	59	5.83	144.3	69.3	109.2	77.2
280	O00410	Importin-5 [OS=Homo sapiens]	↓	30.871	7	5	123.6	4.94	147.7	80.4	94.2	77.8
281	Q00577	Transcriptional activator protein Pur-alpha [OS=Homo sapiens]	↓	30.615	27	4	34.9	6.44	153.9	61.4	109	75.6
282	P46781	40S ribosomal protein S9 [OS=Homo sapiens]	↑	30.452	37	9	22.6	10.65	50.6	128.7	125.5	95.3
283	O94826	Mitochondrial import receptor subunit TOM70 [OS=Homo sapiens]	↓	30.354	10	5	67.4	7.12	135.1	82.9	97.4	84.6
284	O60437	periplakin [OS=Homo sapiens]	↓	30.15	4	7	204.6	5.6	125.7	62.3	109.1	102.9
285	P05023	Sodium/potassium-transporting ATPase subunit alpha-1 [OS=Homo sap	↓	30.029	7	5	112.8	5.49	109.5	99.8	115.8	74.9
286	P48444	Coatomer subunit delta [OS=Homo sapiens]	↓	30.026	10	5	57.2	6.21	132.9	102.7	92	72.3
287	Q9NVA2-1	Septin-11 [OS=Homo sapiens]	↓	29.777	11	4	49.4	6.81	123.9	116	91.1	69
288	Q9Y696	Chloride intracellular channel protein 4 [OS=Homo sapiens]	↓	29.606	34	6	28.8	5.59	120.8	101.9	96.5	80.7
289	O60832-1	H/ACA ribonucleoprotein complex subunit 4 [OS=Homo sapiens]	↑	29.572	13	4	57.6	9.42	60.7	106.4	130.6	102.3
290	P18621	60S ribosomal protein L17 [OS=Homo sapiens]	↑	29.432	26	4	21.4	10.17	53.8	108.9	115.2	122
291	P61088	ubiquitin-conjugating enzyme E2 N [OS=Homo sapiens]	↓	29.388	48	4	17.1	6.57	148.2	96.9	81.4	73.5
292	Q9NUJ1	Mycophenolic acid acyl-glucuronide esterase, mitochondrial [OS=Homo	↓	28.911	15	4	33.9	8.57	177.4	41.8	98.9	81.9
293	P41252	isoleucine--tRNA ligase, cytoplasmic [OS=Homo sapiens]	↓	28.656	6	7	144.4	6.15	172.5	65.3	112.7	49.5
294	P02787	Serotransferrin [OS=Homo sapiens]	↑	28.58	2	2	77	7.12	88.2	150.4	86.1	75.3
295	P62873	Guanine nucleotide-binding protein G(I)/G(S)/G(T) subunit beta-1 [OS=H	↓	28.576	19	5	37.4	6	137.3	93.9	92.9	75.9
296	P62910	60S ribosomal protein L32 [OS=Homo sapiens]	↑	28.359	28	3	15.9	11.33	34	118.2	108.2	139.6
297	Q02543	60S ribosomal protein L18a [OS=Homo sapiens]	↑	28.235	30	5	20.7	10.71	23.9	128.2	122.6	125.3
298	Q8TD30-1	Alanine aminotransferase 2 [OS=Homo sapiens]	↓	28.143	16	5	57.9	7.71	121.9	78.3	110.5	89.3
299	P11387	DNA topoisomerase 1 [OS=Homo sapiens]	↑	28.122	12	7	90.7	9.31	60.3	121.6	120.5	97.6
300	Q15717	ELAV-like protein 1 [OS=Homo sapiens]	↓	28.101	15	4	36.1	9.17	135	88.9	93.6	82.6
301	Q9Y3F4	Serine-threonine kinase receptor-associated protein [OS=Homo sapiens]	↓	27.967	24	6	38.4	5.12	120.9	102.9	98	78.3
302	O00391	Sulfhydryl oxidase 1 [OS=Homo sapiens]	↓	27.847	9	5	82.5	8.92	169.1	103.3	78.4	49.2
303	P27694	Replication protein A 70 kDa DNA-binding subunit [OS=Homo sapiens]	↓	27.431	12	4	68.1	7.21	125.8	89.2	103.2	81.8
304	O43813	LanC-like protein 1 [OS=Homo sapiens]	↑	27.166	16	5	45.3	7.75	70.4	90.8	110.1	128.7
305	Q9UIJ7-1	GTP:AMP phosphotransferase AK3, mitochondrial [OS=Homo sapiens]	↓	27.159	23	4	25.6	9.16	140.3	91.8	89.9	78
306	P51148	Ras-related protein Rab-5C [OS=Homo sapiens]	↓	27.134	22	4	23.5	8.41	122.1	110.1	93.5	74.3
307	Q9UHD8-1	Septin-9 [OS=Homo sapiens]	↓	27.123	10	5	65.4	8.97	136.5	95.6	97.5	70.4
308	P32969	60S ribosomal protein L9 [OS=Homo sapiens]	↓	26.537	31	3	21.9	9.95	117.7	100.3	105.3	76.7
309	P62280	40S ribosomal protein S11 [OS=Homo sapiens]	↑	26.452	35	5	18.4	10.3	70.3	108.9	124.1	96.7
310	Q08945	FACT complex subunit SSRP1 [OS=Homo sapiens]	↓	26.392	8	4	81	6.87	139	101.2	90.4	69.4
311	P05976-1	Myosin light chain 1/3, skeletal muscle isoform [OS=Homo sapiens]	↓	26.264	21	5	21.1	5.03	108	45.3	236.8	10
312	P62851	40S ribosomal protein S25 [OS=Homo sapiens]	↑	26.225	30	5	13.7	10.11	63.6	103.7	99.8	132.9
313	P46778	60S ribosomal protein L21 [OS=Homo sapiens]	↑	26.205	21	3	18.6	10.49	37.9	113.6	125.6	122.9
314	P42126	Enoyl-CoA delta isomerase 1, mitochondrial [OS=Homo sapiens]	↓	26.017	17	4	32.8	8.54	147.4	76	94.1	82.5

315	Q5SSJ5-1	Heterochromatin protein 1-binding protein 3 [OS=Homo sapiens]	↑	25.908	10	5	61.2	9.67	45.7	108.7	137.5	108.1
316	P35998	26S proteasome regulatory subunit 7 [OS=Homo sapiens]	↓	25.881	11	4	48.6	5.95	163.1	79.7	89.6	67.7
317	P62333	26S proteasome regulatory subunit 10B [OS=Homo sapiens]	↓	25.677	13	4	44.1	7.49	151.1	82.9	91.3	74.6
318	Q96RQ3	Methylcrotonoyl-CoA carboxylase subunit alpha, mitochondrial [OS=Hor	↓	25.58	7	4	80.4	7.78	134.6	65.1	110.8	89.6
319	O60763-1	General vesicular transport factor p115 [OS=Homo sapiens]	↓	25.358	6	5	107.8	4.91	130.9	84.5	97.4	87.3
320	Q96C86	M7GpppX diphosphatase [OS=Homo sapiens]	↓	25.329	19	4	38.6	6.38	144.4	97.8	90.5	67.2
321	O15145	Actin-related protein 2/3 complex subunit 3 [OS=Homo sapiens]	↓	25.119	28	5	20.5	8.59	123.9	98.9	96.9	80.4
322	Q7L2H7	Eukaryotic translation initiation factor 3 subunit M [OS=Homo sapiens]	↓	24.91	24	5	42.5	5.63	167.6	79.5	83.8	69.2
323	O94874-1	E3 UFM1-protein ligase 1 [OS=Homo sapiens]	↓	24.812	7	4	89.5	6.79	150.2	86.4	109.5	53.9
324	O00629	Importin subunit alpha-3 [OS=Homo sapiens]	↓	24.792	11	3	57.9	4.96	136.2	102.8	93.1	67.9
325	Q9HC35	Echinoderm microtubule-associated protein-like 4 [OS=Homo sapiens]	↓	24.753	6	5	108.8	6.4	117.4	100.3	105.5	76.8
326	P54725-1	UV excision repair protein RAD23 homolog A [OS=Homo sapiens]	↓	24.731	21	4	39.6	4.58	132.1	115.1	86.2	66.6
327	P09327	Villin-1 [OS=Homo sapiens]	↓	24.685	10	5	92.6	6.39	144.9	68.1	106.1	80.9
328	P59998	Actin-related protein 2/3 complex subunit 4 [OS=Homo sapiens]	↓	24.667	36	5	19.7	8.43	111.3	112.2	104.3	72.2
329	Q5R3I4	tetratricopeptide repeat protein 38 [OS=Homo sapiens]	↓	24.649	12	5	52.8	5.99	126.4	88	115.5	70.2
330	O96008	Mitochondrial import receptor subunit TOM40 homolog [OS=Homo sap	↓	24.331	12	3	37.9	7.25	155.3	96.4	107.6	40.6
331	Q07157	Tight junction protein ZO-1 [OS=Homo sapiens]	↓	24.326	3	4	195.3	6.7	146	93.3	90	70.7
332	P20674	Cytochrome c oxidase subunit 5A, mitochondrial [OS=Homo sapiens]	↓	24.322	47	5	16.8	6.79	167.2	65.3	103	64.4
333	P56470	galectin-4 [OS=Homo sapiens]	↓	24.294	20	4	35.9	9.16	131.5	108.7	88.7	71.2
334	P07305	Histone H1.0 [OS=Homo sapiens]	↑	24.268	21	4	20.9	10.84	18.2	104.6	117.7	159.4
335	Q8WW12	PEST proteolytic signal-containing nuclear protein [OS=Homo sapiens]	↓	24.215	15	2	18.9	7.49	123.5	100.4	92.2	83.9
336	P07451	carbonic anhydrase 3 [OS=Homo sapiens]	↓	24.066	17	3	29.5	7.34	52.8	40.8	274.8	31.6
337	P67870	Casein kinase II subunit beta [OS=Homo sapiens]	↓	23.86	27	4	24.9	5.55	114.4	106.4	76	103.1
338	P78527	DNA-dependent protein kinase catalytic subunit [OS=Homo sapiens]	↓	23.796	1	5	468.8	7.12	98.7	82.4	135.2	83.8
339	P00450	Ceruloplasmin [OS=Homo sapiens]	↑	23.73	6	4	122.1	5.72	0.6	201.8	57.1	140.4
340	O95299	NADH dehydrogenase [ubiquinone] 1 alpha subcomplex subunit 10, mit	↓	23.442	19	4	40.7	8.48	143.2	77.5	98.8	80.5
341	Q9NR45	sialic acid synthase [OS=Homo sapiens]	↓	22.979	8	2	40.3	6.74	152.5	97.7	92.8	57
342	Q9HB71	Calcyclin-binding protein [OS=Homo sapiens]	↓	22.966	14	2	26.2	8.25	129.2	87.1	101.5	82.2
343	P02679	Fibrinogen gamma chain [OS=Homo sapiens]	↑	22.923	9	3	51.5	5.62	66.2	167.5	63.6	102.7
344	O00754	Lysosomal alpha-mannosidase [OS=Homo sapiens]	↑	22.829	4	3	113.7	7.28	43.2	120.4	119.5	116.8
345	O43583	density-regulated protein [OS=Homo sapiens]	↑	22.713	26	4	22.1	5.3	76.5	91.8	121	110.7
346	P42025	Beta-centractin [OS=Homo sapiens]	↑	22.679	21	4	42.3	6.4	4.8	250.7	68.3	76.3
347	P35611-1	Alpha-adducin [OS=Homo sapiens]	↑	22.647	8	3	80.9	5.83	113.3	151.7	11.9	123.1
348	P45378-1	Troponin T, fast skeletal muscle [OS=Homo sapiens]	↑	22.611	19	4	31.8	5.9	3.5	255.8	124.5	16.2
349	Q8N8S7	Protein enabled homolog [OS=Homo sapiens]	↓	22.524	9	5	66.5	6.93	108.1	70.4	115.9	105.5
350	Q9BY43	Charged multivesicular body protein 4a [OS=Homo sapiens]	↑	22.434	23	4	25.1	4.7	62.1	87.2	106.9	143.7
351	Q9Y266	nuclear migration protein nudC [OS=Homo sapiens]	↓	22.38	17	5	38.2	5.38	133.7	87.9	98.9	79.6
352	Q9H9B4	Sideroflexin-1 [OS=Homo sapiens]	↓	22.357	18	3	35.6	9.07	136.1	89.7	92.8	81.4
353	Q04446	1,4-alpha-glucan-branching enzyme [OS=Homo sapiens]	↑	22.243	11	5	80.4	6.32	45	102.3	125.1	127.6
354	P17661	desmin [OS=Homo sapiens]	↑	22.018	8	5	53.5	5.27	88.6	94	123.7	93.8
355	O60884	DnaJ homolog subfamily A member 2 [OS=Homo sapiens]	↑	22.001	17	3	45.7	6.48	40.5	163.7	39.4	156.4
356	Q9Y365	START domain-containing protein 10 [OS=Homo sapiens]	↓	21.887	14	3	33	7.12	170.9	74.5	98.7	55.9
357	Q13308-1	Inactive tyrosine-protein kinase 7 [OS=Homo sapiens]	↓	21.731	5	4	118.3	7.09	131.7	85.7	97.2	85.5
358	P13861	cAMP-dependent protein kinase type II-alpha regulatory subunit [OS=H	↓	21.638	11	3	45.5	5.07	189.1	18.1	107.4	85.4
359	O95433	activator of 90 kDa heat shock protein ATPase homolog 1 [OS=Homo s	↓	21.613	13	3	38.3	5.53	160	73.5	99.5	67.1
360	P06454-1	Prothymosin alpha [OS=Homo sapiens]	↑	21.547	22	3	12.2	3.78	79	111.5	126	83.5
361	P11908	ribose-phosphate pyrophosphokinase 2 [OS=Homo sapiens]	↓	21.529	19	4	34.7	6.61	180.4	113.3	78	28.4
362	P08134	Rho-related GTP-binding protein RhoC [OS=Homo sapiens]	↓	21.414	25	4	22	6.58	131.5	104.7	88.7	75.1
363	P61586	Transforming protein RhoA [OS=Homo sapiens]	↓	21.297	25	4	21.8	6.1	128.1	106.2	87.2	78.5
364	Q9P2M7-1	cingulin [OS=Homo sapiens]	↓	21.156	6	5	136.3	5.54	120.6	82.5	104.2	92.8
365	Q9Y314	nitric oxide synthase-interacting protein [OS=Homo sapiens]	↑	21.142	20	4	33.2	8.82	67.7	73.2	94.8	164.4
366	P42766	60S ribosomal protein L35 [OS=Homo sapiens]	↑	21.062	26	3	14.5	11.05	31.7	96.4	122.5	149.4
367	P51571	translocon-associated protein subunit delta [OS=Homo sapiens]	↓	20.926	25	3	19	6.15	131.3	95.1	101.3	72.4
368	O43488	aflatoxin B1 aldehyde reductase member 2 [OS=Homo sapiens]	↓	20.773	13	3	39.6	7.17	159.5	76	90.5	74
369	P22102-1	trifunctional purine biosynthetic protein adenosine-3 [OS=Homo sapiens	↓	20.719	8	5	107.7	6.7	160.6	60.3	134.9	44.2
370	Q8N163-1	Cell cycle and apoptosis regulator protein 2 [OS=Homo sapiens]	↓	20.548	7	4	102.8	5.22	171.9	86.7	88	53.4
371	Q15257-1	Serine/threonine-protein phosphatase 2A activator [OS=Homo sapiens]	↓	20.524	17	3	40.6	5.94	131.5	92.2	95.6	80.7
372	Q14498-1	RNA-binding protein 39 [OS=Homo sapiens]	↓	20.455	10	4	59.3	10.1	148.8	80.4	93.4	77.4
373	P30711	Glutathione S-transferase theta-1 [OS=Homo sapiens]	↓	20.271	19	3	27.3	7.49	144.2	80.8	97.1	77.9
374	P51608	Methyl-CpG-binding protein 2 [OS=Homo sapiens]	↑	20.222	7	2	52.4	9.95	59.6	116.2	97.1	127
375	Q9Y3U8	60S ribosomal protein L36 [OS=Homo sapiens]	↑	20.21	30	4	12.2	11.59	35.9	118.8	120.2	125
376	P12081	Histidine--tRNA ligase, cytoplasmic [OS=Homo sapiens]	↓	20.133	8	4	57.4	5.88	137.1	98.2	98.5	66.3
377	Q13642-1	Isoform 1 of Four and a half LIM domains protein 1 [OS=Homo sapiens	↑	20.042	21	5	31.9	8.32	87.1	55.7	199.9	57.3

378	O00584	Ribonuclease T2 [OS=Homo sapiens]	↑	20.021	23	4	29.5	7.08	61.9	104.5	119.6	114.1
379	P54802	alpha-N-acetylglucosaminidase [OS=Homo sapiens]	↑	20.017	11	5	82.2	6.65	74.4	115.7	101.6	108.4
380	Q96EK6	glucosamine 6-phosphate N-acetyltransferase [OS=Homo sapiens]	↓	19.976	33	3	20.7	7.99	135.1	109.3	90.9	64.7
381	Q9NUQ9	protein FAM49B [OS=Homo sapiens]	↓	19.852	13	3	36.7	6.06	130.3	97.9	97.7	74.1
382	P63173	60s ribosomal protein l38 [OS=Homo sapiens]	↓	19.826	36	3	8.2	10.1	126.7	81.5	104.9	86.8
383	O95479	GDH/6PGL endoplasmic bifunctional protein [OS=Homo sapiens]	↓	19.675	5	3	88.8	7.3	143.9	82.9	99.6	73.6
384	P26373-1	60S ribosomal protein L13 [OS=Homo sapiens]	↑	19.421	25	5	24.2	11.65	30.6	134.9	123.8	110.7
385	P04083	annexin A1 [OS=Homo sapiens]	↑	19.279	9	3	38.7	7.02	64.8	159.9	86.9	88.3
386	P23381	Tryptophan--tRNA ligase, cytoplasmic [OS=Homo sapiens]	↓	19.219	9	3	53.1	6.23	162.3	83.5	86	68.3
387	Q9UHB6	LIM domain and actin-binding protein 1 [OS=Homo sapiens]	↓	19.208	6	3	85.2	6.84	164.6	51.7	103.3	80.5
388	P24821	Tenascin [OS=Homo sapiens]	↑	19.055	2	4	240.7	4.89	29.3	153.7	30.1	187
389	P40429	60S ribosomal protein L13a [OS=Homo sapiens]	↑	18.961	23	5	23.6	10.93	8.4	110.5	129.1	152
390	Q92769	Histone deacetylase 2 [OS=Homo sapiens]	↓	18.941	15	4	55.3	5.91	127.9	91.9	93.5	86.7
391	Q08170	Serine/arginine-rich splicing factor 4 [OS=Homo sapiens]	↓	18.902	9	5	56.6	11.52	137.7	55.5	96.3	110.5
392	P31483	nucleolysin TIA-1 isoform p40 [OS=Homo sapiens]	↓	18.882	8	2	42.9	7.74	136	90.9	91.8	81.3
393	P35270	Sepiapterin reductase [OS=Homo sapiens]	↓	18.856	20	3	28	8.05	150.4	59.6	120.1	69.9
394	P19784	casein kinase II subunit alpha' [OS=Homo sapiens]	↑	18.798	12	3	41.2	8.56	55.9	146.6	87	110.5
395	P24539	ATP synthase F(0) complex subunit B1, mitochondrial [OS=Homo sapier	↓	18.709	23	4	28.9	9.36	130.9	74.9	102.9	91.2
396	Q9BYZ2	L-lactate dehydrogenase A-like 6B [OS=Homo sapiens]	↑	18.635	9	2	41.9	8.65	69.2	141.1	90.2	99.4
397	P08263	Glutathione S-transferase A1 [OS=Homo sapiens]	↓	18.342	17	4	25.6	8.88	111.7	77.8	113.3	97.2
398	P13987	CD59 glycoprotein [OS=Homo sapiens]	↓	18.286	25	3	14.2	6.48	135	77.4	96.8	90.8
399	P46459	Vesicle-fusing ATPase [OS=Homo sapiens]	↓	18.199	10	5	82.5	6.95	152.4	88.2	91.8	67.6
400	P20339	Ras-related protein Rab-5A [OS=Homo sapiens]	↓	18.067	17	3	23.6	8.15	130	105.2	88.8	75.9
401	O75608	Acyl-protein thioesterase 1 [OS=Homo sapiens]	↓	18.021	24	4	24.7	6.77	154.2	91.7	83.5	70.5
402	P52788-1	Spermine synthase [OS=Homo sapiens]	↓	18.019	24	4	41.2	5.02	177	61.3	91.5	70.2
403	O75821	Eukaryotic translation initiation factor 3 subunit G [OS=Homo sapiens]	↓	17.768	12	3	35.6	6.13	124.6	92.9	101.7	80.9
404	O00264	Membrane-associated progesterone receptor component 1 [OS=Homo	↓	17.587	23	2	21.7	4.7	132.8	93	99.1	75
405	Q9UNH7-1	Sorting nexin-6 [OS=Homo sapiens]	↓	17.529	10	4	46.6	6.16	135.2	94.8	91.1	78.8
406	P02794	Ferritin heavy chain [OS=Homo sapiens]	↑	17.506	21	4	21.2	5.55	73.2	141.8	91.6	93.4
407	Q92522	Histone H1x [OS=Homo sapiens]	↑	17.406	15	3	22.5	10.76	34.6	103.9	120.2	141.2
408	P46379-1	Large proline-rich protein BAG6 [OS=Homo sapiens]	↓	17.401	5	3	119.3	5.6	162.7	83.8	56.9	96.6
409	P06132	Uroporphyrinogen decarboxylase [OS=Homo sapiens]	↓	17.36	18	3	40.8	6.14	134.8	81.6	113.9	69.7
410	Q9NYL9	tropomodulin-3 [OS=Homo sapiens]	↑	17.29	14	3	39.6	5.19	77.3	53.1	125.6	144
411	Q86X29	Lipolysis-stimulated lipoprotein receptor [OS=Homo sapiens]	↓	17.222	4	2	71.4	7.97	129.7	94.8	93.6	81.8
412	Q8IX12	Cell division cycle and apoptosis regulator protein 1 [OS=Homo sapiens	↓	17.113	3	2	132.7	5.76	131.6	106.5	89.2	72.7
413	Q9UBL3-1	Set1/Ash2 histone methyltransferase complex subunit ASH2 [OS=Homo	↓	17.013	8	3	68.7	5.69	132	103.3	115.6	49
414	P15586	N-acetylglucosamine-6-sulfatase [OS=Homo sapiens]	↓	16.961	10	3	62	8.31	134.9	96.2	100.6	68.3
415	Q969G3	SWI/SNF-related matrix-associated actin-dependent regulator of chroma	↑	16.711	9	3	46.6	4.88	59.5	124.3	116.3	99.8
416	P84098	60S ribosomal protein L19 [OS=Homo sapiens]	↑	16.551	18	3	23.5	11.47	11.9	128.4	129.9	129.8
417	Q8WW59	SPRY domain-containing protein 4 [OS=Homo sapiens]	↓	16.535	28	3	23.1	6.93	112.6	83.1	104.8	99.5
418	P14091	Cathepsin E [OS=Homo sapiens]	↓	16.331	9	3	43.3	4.86	132.8	99.8	90.9	76.5
419	O43747-1	AP-1 complex subunit gamma-1 [OS=Homo sapiens]	↓	16.268	8	4	91.3	6.8	115.6	104.5	109	70.9
420	O94992	Protein HEXIM1 [OS=Homo sapiens]	↑	16.249	9	2	40.6	4.89	77	106.8	105.3	110.9
421	P63000-1	Ras-related C3 botulinum toxin substrate 1 [OS=Homo sapiens]	↓	15.959	29	5	21.4	8.5	170.1	86	94.3	49.7
422	P48556	26S proteasome non-ATPase regulatory subunit 8 [OS=Homo sapiens]	↓	15.922	9	3	39.6	9.7	115.4	95.3	113.7	75.6
423	P10619	lysosomal protective protein [OS=Homo sapiens]	↓	15.891	5	2	54.4	6.61	142.2	95.3	86.4	76.1
424	P56134	ATP synthase subunit f, mitochondrial [OS=Homo sapiens]	↑	15.837	26	2	10.9	9.67	71.5	102.4	104.7	121.5
425	P15170-3	Isoform 3 of Eukaryotic peptide chain release factor GTP-binding subun	↓	15.769	5	3	68.7	5.33	139.1	87.5	93.5	79.8
426	Q14204	Cytoplasmic dynein 1 heavy chain 1 [OS=Homo sapiens]	↓	15.749	2	4	532.1	6.4	133.6	75.5	107.3	83.6
427	P61086	Ubiquitin-conjugating enzyme E2 K [OS=Homo sapiens]	↓	15.713	29	3	22.4	5.44	135.5	83.8	100	80.7
428	Q3LXA3	Triokinase/FMN cyclase [OS=Homo sapiens]	↓	15.627	5	2	58.9	7.49	137.4	74.4	103.2	85
429	Q9H8Y8-1	Golgi reassembly-stacking protein 2 [OS=Homo sapiens]	↓	15.536	7	2	47.1	4.82	145.4	77.4	104	73.2
430	Q04323-1	UBX domain-containing protein 1 [OS=Homo sapiens]	↓	15.472	10	2	33.3	5.25	132.1	121.5	32.2	114.1
431	O60343-1	TBC1 domain family member 4 [OS=Homo sapiens]	↓	15.459	3	3	146.5	7.01	121.1	80.5	118.3	80.2
432	Q9UHN6-1	Cell surface hyaluronidase [OS=Homo sapiens]	↓	15.441	6	4	154.3	8.15	121.2	94.2	112.9	71.7
433	Q13404	Ubiquitin-conjugating enzyme E2 variant 1 [OS=Homo sapiens]	↓	15.329	24	4	16.5	7.93	156.7	82.6	91.6	69
434	Q9NSD9	Phenylalanine--tRNA ligase beta subunit [OS=Homo sapiens]	↓	15.226	6	4	66.1	6.84	190.3	98.5	68.4	42.8
435	Q8NFH4	Nucleoporin Nup37 [OS=Homo sapiens]	↑	15.129	20	4	36.7	5.92	59	93.5	119.2	128.2
436	P49959	Double-strand break repair protein MRE11 [OS=Homo sapiens]	↓	15.118	8	3	80.5	5.9	174.1	84	86.3	55.6
437	B5ME19	eukaryotic translation initiation factor 3 subunit C-like protein [OS=Hom	↓	15.108	4	3	105.4	5.64	114.5	106	105.1	74.4
438	P61019-1	Ras-related protein Rab-2A [OS=Homo sapiens]	↓	14.999	13	2	23.5	6.54	121.7	102.5	95.7	80.1
439	Q8N684-1	Cleavage and polyadenylation specificity factor subunit 7 [OS=Homo sa	↓	14.937	8	2	52	8	180.4	44.9	42.1	132.7
440	O96019-1	Actin-like protein 6A [OS=Homo sapiens]	↓	14.69	9	2	47.4	5.6	132.3	105.3	93.4	68.9

441	P46779	60S ribosomal protein L28 [OS=Homo sapiens]	↑	14.688	22	3	15.7	12.02	36.8	110	121.4	131.7
442	P01024	Complement C3 [OS=Homo sapiens]	↑	14.67	2	3	187	6.4	49.4	102.4	117.5	130.7
443	P22352	Glutathione peroxidase 3 [OS=Homo sapiens]	↑	14.553	14	3	25.5	8.13	51.9	159	74	115.1
444	Q13564-1	NEDD8-activating enzyme E1 regulatory subunit [OS=Homo sapiens]	↓	14.517	10	3	60.2	5.4	145.9	90.6	90.5	73
445	O95861-1	3'(2'),5'-bisphosphate nucleotidase 1 [OS=Homo sapiens]	↓	14.514	20	3	33.4	5.69	153.3	77.3	92.5	76.9
446	Q9UN86	Ras GTPase-activating protein-binding protein 2 [OS=Homo sapiens]	↓	14.388	6	2	54.1	5.55	106.8	96.9	103.5	92.7
447	P46776	60S ribosomal protein L27a [OS=Homo sapiens]	↑	14.378	20	3	16.6	11	58.2	124.8	121	96
448	Q9NXG2	THUMP domain-containing protein 1 [OS=Homo sapiens]	↓	14.186	7	2	39.3	7.88	132.2	95.3	97.5	75
449	O14980	Exportin-1 [OS=Homo sapiens]	↓	14.178	4	3	123.3	6.06	123.8	71.7	107.2	97.4
450	P61513	60S ribosomal protein L37a [OS=Homo sapiens]	↑	14.16	41	3	10.3	10.43	54.5	123.4	120.9	101.2
451	Q00325-2	Isoform B of Phosphate carrier protein, mitochondrial [OS=Homo sapier	↓	13.953	15	4	39.9	9.36	103.6	74.8	131.7	89.9
452	P20774	Mimecan [OS=Homo sapiens]	↑	13.899	6	2	33.9	5.63	20.9	39.9	294.9	44.2
453	Q92900	Regulator of nonsense transcripts 1 [OS=Homo sapiens]	↓	13.886	4	4	124.3	6.61	139.8	96.3	93.8	70.1
454	P53990-1	IST1 homolog [OS=Homo sapiens]	↑	13.868	10	3	39.7	5.35	80.6	99.4	115.2	104.8
455	P61923	Coatomer subunit zeta-1 [OS=Homo sapiens]	↓	13.696	16	2	20.2	4.81	146.1	108.1	100.5	45.3
456	Q8TBC4-1	NEDD8-activating enzyme E1 catalytic subunit [OS=Homo sapiens]	↑	13.62	11	3	51.8	5.45	60	112.9	109.9	117.2
457	Q9H4A6	Golgi phosphoprotein 3 [OS=Homo sapiens]	↓	13.581	18	3	33.8	6.44	199.3	72	65.9	62.8
458	P08195	4F2 cell-surface antigen heavy chain [OS=Homo sapiens]	↓	13.572	4	2	68	5.01	151.5	95.8	102.9	49.8
459	Q13557	Calcium/calmodulin-dependent protein kinase type II subunit delta [OS=	↓	13.297	8	3	56.3	7.25	135.1	86	102.4	76.5
460	P61026	ras-related protein rab-10 [OS=Homo sapiens]	↓	13.179	15	3	22.5	8.38	126.4	124.1	84	65.5
461	P49915	GMP synthase [glutamine-hydrolyzing] [OS=Homo sapiens]	↓	13.029	5	3	76.7	6.87	155.2	101.3	102.5	41
462	Q01581	hydroxymethylglutaryl-CoA synthase, cytoplasmic [OS=Homo sapiens]	↓	13.013	7	3	57.3	5.41	114.2	79.3	126.8	79.7
463	Q8WVM8	Sec1 family domain-containing protein 1 [OS=Homo sapiens]	↓	12.965	5	2	72.3	6.27	160.9	85.6	87.5	66
464	P02689	Myelin P2 protein [OS=Homo sapiens]	↑	12.927	14	2	14.9	9.83	77	144.7	101.5	76.8
465	Q05519	serine/arginine-rich splicing factor 11 [OS=Homo sapiens]	↓	12.904	8	2	53.5	10.52	162	82.6	59.3	96.2
466	O43237	Cytoplasmic dynein 1 light intermediate chain 2 [OS=Homo sapiens]	↓	12.894	16	3	54.1	6.38	154.6	97.5	84.4	63.4
467	P02790	Hemopexin [OS=Homo sapiens]	↑	12.664	7	4	51.6	7.02	75.9	142.5	82.4	99.2
468	P17480-1	Nucleolar transcription factor 1 [OS=Homo sapiens]	↑	12.662	5	3	89.4	5.81	64.5	108.8	107.4	119.3
469	Q9NZL4-1	Hsp70-binding protein 1 [OS=Homo sapiens]	↓	12.642	10	2	39.4	5.21	140.6	57.9	108.8	92.6
470	P08559	Pyruvate dehydrogenase E1 component subunit alpha, somatic form, m	↓	12.483	14	3	43.3	8.06	123.6	94.6	99.9	81.9
471	P61201-1	COP9 signalosome complex subunit 2 [OS=Homo sapiens]	↑	12.353	9	2	51.6	5.53	46.9	124.3	134.9	93.9
472	O43823	A-kinase anchor protein 8 [OS=Homo sapiens]	↓	12.344	5	2	76.1	5.15	136.2	76.1	102.7	85
473	O75251	NADH dehydrogenase [ubiquinone] iron-sulfur protein 7, mitochondrial	↓	12.305	17	2	23.5	9.99	140.4	83	106.4	70.2
474	Q08752	peptidyl-prolyl cis-trans isomerase D [OS=Homo sapiens]	↑	12.259	9	3	40.7	7.21	56.7	121.6	110.5	111.2
475	Q9BRP8	Partner of Y14 and mago [OS=Homo sapiens]	↑	12.221	14	2	22.6	9.45	84.5	74.9	128.1	112.5
476	Q9UQE7	Structural maintenance of chromosomes protein 3 [OS=Homo sapiens]	↓	12.114	2	2	141.5	7.18	149.7	99.7	83.6	67
477	P14555	Phospholipase A2, membrane associated [OS=Homo sapiens]	↑	11.93	17	2	16.1	9.23	20.1	31.6	29.9	318.4
478	P13674-1	prolyl 4-hydroxylase subunit alpha-1 [OS=Homo sapiens]	↓	11.862	5	2	61	6.01	139.9	120.3	50.7	89.1
479	Q13084	39S ribosomal protein L28, mitochondrial [OS=Homo sapiens]	↓	11.84	16	3	30.1	8.29	107.8	90.7	85.6	115.9
480	Q9Y262	eukaryotic translation initiation factor 3 subunit L [OS=Homo sapiens]	↓	11.8	5	2	66.7	6.34	197.8	72.1	72.4	57.7
481	Q9BXP5	serrate RNA effector molecule homolog [OS=Homo sapiens]	↓	11.799	7	2	100.6	5.96	128.7	72.8	104.4	94.1
482	Q9H444	Charged multivesicular body protein 4b [OS=Homo sapiens]	↑	11.793	11	2	24.9	4.82	77.7	95.3	102.9	124.1
483	Q8NE71-1	ATP-binding cassette sub-family F member 1 [OS=Homo sapiens]	↓	11.694	3	2	95.9	6.8	138.5	75.2	113.7	72.7
484	P12111	Collagen alpha-3(VI) chain [OS=Homo sapiens]	↑	11.678	1	2	343.5	6.68	52.3	167.2	81.1	99.4
485	Q02809	Procollagen-lysine,2-oxoglutarate 5-dioxygenase 1 [OS=Homo sapiens]	↓	11.617	4	2	83.5	6.95	135	100.4	90	74.6
486	Q2TAA2	Isoamyl acetate-hydrolyzing esterase 1 homolog [OS=Homo sapiens]	↓	11.59	11	2	27.6	5.3	141	93.9	97.2	67.9
487	P07947	tyrosine-protein kinase Yes [OS=Homo sapiens]	↓	11.589	5	3	60.8	6.74	150.2	81.4	96.8	71.5
488	Q9UBF2	Coatomer subunit gamma-2 [OS=Homo sapiens]	↓	11.582	4	2	97.6	5.81	138.7	92.7	107.3	61.3
489	Q9BVK6	Transmembrane emp24 domain-containing protein 9 [OS=Homo sapien	↓	11.525	9	3	27.3	8.02	189.3	45.9	131.7	33.1
490	Q14978-1	nucleolar and coiled-body phosphoprotein 1 [OS=Homo sapiens]	↑	11.515	4	2	73.6	9.47	64.1	98.1	130.9	106.8
491	P13807	glycogen [starch] synthase, muscle [OS=Homo sapiens]	↑	11.507	4	2	83.7	6.18	60.8	117.3	151.4	70.5
492	Q9NR31	GTP-binding protein SAR1a [OS=Homo sapiens]	↓	11.448	12	2	22.4	6.68	140.5	100	87.2	72.2
493	Q16698	2,4-dienoyl-CoA reductase, mitochondrial [OS=Homo sapiens]	↓	11.376	8	3	36	9.28	202.2	60.6	76.9	60.4
494	P00488	Coagulation factor XIII A chain [OS=Homo sapiens]	↑	11.305	4	3	83.2	6.09	69.5	144.6	75.7	110.3
495	Q9Y2W2	WW domain-binding protein 11 [OS=Homo sapiens]	↑	11.21	5	3	70	8.38	58	60.6	178.1	103.3
496	P51884	Lumican [OS=Homo sapiens]	↑	11.136	6	2	38.4	6.61	54.7	171.6	82.9	90.8
497	P15374	Ubiquitin carboxyl-terminal hydrolase isozyme L3 [OS=Homo sapiens]	↑	11.079	11	2	26.2	4.92	116	115.9	93.4	74.7
498	O95394	phosphoacetylglucosamine mutase [OS=Homo sapiens]	↓	10.96	9	2	59.8	6.25	145.2	81.3	98.3	75.1
499	O75306-1	NADH dehydrogenase [ubiquinone] iron-sulfur protein 2, mitochondrial	↓	10.931	6	3	52.5	7.55	149.5	73	103.7	73.8
500	P21810	biglycan [OS=Homo sapiens]	↑	10.923	7	2	41.6	7.52	63.9	143.1	87.1	105.9
501	O95571	Persulfide dioxygenase ETHE1, mitochondrial [OS=Homo sapiens]	↓	10.906	11	2	27.9	6.83	137.9	96	86.2	80
502	P13073	Cytochrome c oxidase subunit 4 isoform 1, mitochondrial [OS=Homo sa	↓	10.833	20	3	19.6	9.51	139.4	70.3	102.1	88.2
503	Q9Y277	Voltage-dependent anion-selective channel protein 3 [OS=Homo sapien	↓	10.816	9	2	30.6	8.66	102.8	78.2	109.8	109.2

504	O00487	26S proteasome non-ATPase regulatory subunit 14 [OS=Homo sapiens]	↓	10.721	15	2	34.6	6.52	141.1	93.6	91.1	74.2
505	O60716-1	Catenin delta-1 [OS=Homo sapiens]	↓	10.72	4	3	108.1	6.23	134.7	92.2	98.4	74.7
506	Q9NNW7-1	thioredoxin reductase 2, mitochondrial [OS=Homo sapiens]	↑	10.698	6	2	56.5	7.5	69.2	111.3	99.1	120.4
507	P19404	NADH dehydrogenase [ubiquinone] flavoprotein 2, mitochondrial [OS=H	↓	10.676	10	2	27.4	8.06	161.7	54.7	145.9	37.8
508	Q99460	26S proteasome non-ATPase regulatory subunit 1 [OS=Homo sapiens]	↓	10.645	4	3	105.8	5.39	144.2	106.6	80.3	68.9
509	Q8NBX0	saccharopine dehydrogenase-like oxidoreductase [OS=Homo sapiens]	↑	10.625	8	3	47.1	9.14	87.6	160.1	85.3	67.1
510	P25189	Myelin protein P0 [OS=Homo sapiens]	↑	10.623	12	3	27.5	9.54	67.5	73	100.3	159.2
511	O75822	Eukaryotic translation initiation factor 3 subunit J [OS=Homo sapiens]	↑	10.619	12	3	29	4.83	85	145.7	84.2	85.1
512	P49792	E3 SUMO-protein ligase RanBP2 [OS=Homo sapiens]	↓	10.564	2	2	358	6.2	131.8	83.5	87.5	97.3
513	O94903	Pyridoxal phosphate homeostasis protein [OS=Homo sapiens]	↓	10.472	8	2	30.3	7.5	155	71.8	105.8	67.5
514	O60256-1	Phosphoribosyl pyrophosphate synthase-associated protein 2 [OS=Hom	↓	10.432	7	2	40.9	7.44	146.7	107.7	84.9	60.7
515	O75947-1	ATP synthase subunit d, mitochondrial [OS=Homo sapiens]	↓	10.295	16	2	18.5	5.3	118.3	74.6	98.7	108.5
516	P49903-1	Selenide, water dikinase 1 [OS=Homo sapiens]	↓	10.229	7	2	42.9	5.97	102	98.8	100.2	98.9
517	O60613-1	selenoprotein F [OS=Homo sapiens]	↓	10.176	18	2	17.8	5.03	131.4	94.7	99.5	74.4
518	P20142	Gastricsin [OS=Homo sapiens]	↓	9.912	5	2	42.4	4.46	180	83.3	81.3	55.3
519	P62495	Eukaryotic peptide chain release factor subunit 1 [OS=Homo sapiens]	↓	9.876	9	2	49	5.71	138	99.2	87.1	75.7
520	P61970	nuclear transport factor 2 [OS=Homo sapiens]	↓	9.799	19	2	14.5	5.38	136.9	85.7	92.8	84.6
521	P13688-1	Carcinoembryonic antigen-related cell adhesion molecule 1 [OS=Homo	↑	9.647	7	2	57.5	5.97	78.4	101	103	117.6
522	Q9UJS0	Calcium-binding mitochondrial carrier protein Aralar2 [OS=Homo sapier	↓	9.521	4	2	74.1	8.62	131.9	82	104.8	81.3
523	O14841	5-oxoprolinase [OS=Homo sapiens]	↓	9.423	3	2	137.4	6.58	148.9	116.2	81.5	53.4
524	P36952-1	Serpin B5 [OS=Homo sapiens]	↓	9.288	9	2	42.1	6.05	126.2	105.8	77.8	90.1
525	Q93009	Ubiquitin carboxyl-terminal hydrolase 7 [OS=Homo sapiens]	↓	9.261	3	3	128.2	5.55	209.8	71.5	79.5	39.2
526	P63151	Serine/threonine-protein phosphatase 2A 55 kDa regulatory subunit B a	↑	9.207	5	2	51.7	6.2	83.9	97.5	123.2	95.4
527	P26640	Valine--tRNA ligase [OS=Homo sapiens]	↓	9.121	5	2	140.4	7.59	182.8	177	19.8	20.4
528	P53992	Protein transport protein sec24c [OS=Homo sapiens]	↓	9.109	3	2	118.2	7.06	218.5	105.5	43.7	32.4
529	P21980	Protein-glutamine gamma-glutamyltransferase 2 [OS=Homo sapiens]	↓	8.98	5	2	77.3	5.22	147	106.6	85.4	61.1
530	P84095	Rho-related GTP-binding protein RhoG [OS=Homo sapiens]	↓	8.912	19	2	21.3	8.12	142.9	122.4	85.4	49.2
531	Q9H5N1	rab GTPase-binding effector protein 2 [OS=Homo sapiens]	↓	8.672	4	2	63.5	4.78	121.9	106.7	97.8	73.5
532	Q5VW32	BRO1 domain-containing protein BROX [OS=Homo sapiens]	↓	8.635	11	2	46.4	7.65	120.2	77	116.5	86.4
533	P61077-1	Ubiquitin-conjugating enzyme E2 D3 [OS=Homo sapiens]	↓	8.553	24	2	16.7	7.8	136.2	99.2	84.4	80.2
534	P21741	Midkine [OS=Homo sapiens]	↑	8.548	12	2	15.6	9.79	50.2	119	100.7	130.1
535	O00483	Cytochrome c oxidase subunit NDUFA4 [OS=Homo sapiens]	↓	8.505	22	2	9.4	9.38	152.6	96.9	87.8	62.7
536	P53007	Tricarboxylate transport protein, mitochondrial [OS=Homo sapiens]	↑	8.392	8	2	34	9.89	64.9	115.9	115.8	103.4
537	P53396-1	ATP-citrate synthase [OS=Homo sapiens]	↓	8.314	4	3	120.8	7.33	157.8	83.7	95.4	63.1
538	O00233-1	26S proteasome non-ATPase regulatory subunit 9 [OS=Homo sapiens]	↑	8.3	9	2	24.7	6.95	66.7	108.2	104.2	120.8
539	Q6DD88	Atlastin-3 [OS=Homo sapiens]	↑	8.284	8	2	60.5	5.66	55.1	121.5	124.7	98.8
540	O00159-1	Unconventional myosin-Ic [OS=Homo sapiens]	↑	8.28	3	2	121.6	9.41	73.2	115.8	128.9	82.1
541	P43251	Biotinidase [OS=Homo sapiens]	↑	8.239	4	2	61.1	6.25	57.1	100.5	128.8	113.6
542	P60228	Eukaryotic translation initiation factor 3 subunit E [OS=Homo sapiens]	↓	8.172	4	2	52.2	6.04	143.3	124.6	77.7	54.4
543	Q9Y285	Phenylalanine--tRNA ligase alpha subunit [OS=Homo sapiens]	↓	8.15	6	2	57.5	7.8	141.5	97.1	98.7	62.7
544	Q9Y6Y8	SEC23-interacting protein [OS=Homo sapiens]	↓	8.059	4	2	111	5.54	175.8	81.6	85.8	56.7
545	P08240-1	signal recognition particle receptor subunit alpha [OS=Homo sapiens]	↓	8.057	7	2	69.8	8.95	140.7	31.7	211.3	16.3
546	P49590	probable histidine--tRNA ligase, mitochondrial [OS=Homo sapiens]	↓	8.019	4	2	56.9	8.24	132.9	86.2	99.2	81.8
547	P61006	Ras-related protein Rab-8A [OS=Homo sapiens]	↓	8.012	9	2	23.7	9.07	131.6	77.6	102.4	88.4
548	P52948-1	nuclear pore complex protein Nup98-Nup96 [OS=Homo sapiens]	↓	8.002	1	2	197.5	6.4	165.5	111.2	63	60.4
549	P30837	Aldehyde dehydrogenase X, mitochondrial [OS=Homo sapiens]	↑	7.824	5	2	57.2	6.8	56.6	85.1	114.7	143.5
550	Q9P0J0	NADH dehydrogenase [ubiquinone] 1 alpha subcomplex subunit 13 [OS	↓	7.817	17	2	16.7	8.43	164.1	77.8	96.2	61.9
551	P52888	thimet oligopeptidase [OS=Homo sapiens]	↑	7.637	3	2	78.8	6.05	64.2	115.4	107.7	112.7
552	P19623	spermidine synthase [OS=Homo sapiens]	↓	7.629	6	2	33.8	5.49	153.6	93.4	87.4	65.7
553	P22061	protein-L-isoaspartate(D-aspartate) O-methyltransferase [OS=Homo sap	↓	7.408	10	2	24.6	7.21	167.9	102.9	82.3	46.9
554	Q13177	Serine/threonine-protein kinase PAK 2 [OS=Homo sapiens]	↓	7.403	5	2	58	5.96	153	90.9	89	67.1
555	P18077	60S ribosomal protein L35a [OS=Homo sapiens]	↑	7.315	21	3	12.5	11.06	43.1	113.3	116	127.6
556	P50552	Vasodilator-stimulated phosphoprotein [OS=Homo sapiens]	↓	7.314	6	2	39.8	8.94	123.5	97.9	94.3	84.3
557	O95758-1	Isoform 1 of Polypyrimidine tract-binding protein 3 [OS=Homo sapiens]	↓	7.218	3	2	56.5	9.22	130.2	103.5	95.1	71.1
558	Q9UEY8-1	Gamma-adducin [OS=Homo sapiens]	↓	7.089	3	2	79.1	6.32	118.2	107.3	104.9	69.6
559	P61254	60S ribosomal protein L26 [OS=Homo sapiens]	↑	6.968	17	3	17.2	10.55	34.3	120.3	130.6	114.9
560	Q9UHY1	Nuclear receptor-binding protein [OS=Homo sapiens]	↓	6.945	6	2	59.8	5.08	181.5	86.8	60.1	71.6
561	Q9GZP4	PITH domain-containing protein 1 [OS=Homo sapiens]	↑	6.873	14	2	24.2	5.74	56.1	94.5	85.7	163.7
562	O15164	Transcription intermediary factor 1-alpha [OS=Homo sapiens]	↓	6.798	3	2	116.8	7.11	142.6	66.6	113.2	77.6
563	Q9BZE9	tether containing UBX domain for GLUT4 [OS=Homo sapiens]	↓	6.632	6	2	60.1	6.64	116.4	91.4	104.7	87.6
564	P61457	Pterin-4-alpha-carbinolamine dehydratase [OS=Homo sapiens]	↓	6.489	23	2	12	6.8	161	70.2	99.8	68.9
565	P09001	39S ribosomal protein L3, mitochondrial [OS=Homo sapiens]	↑	6.298	7	2	38.6	9.48	83.7	56	129.6	130.7
566	Q99611	Selenide, water dikinase 2 [OS=Homo sapiens]	↓	6.244	7	2	47.3	5.88	133.2	97.3	89.6	79.8

567	Q96FX7	tRNA (adenine(58)-N(1))-methyltransferase catalytic subunit TRMT61A [(	↓	6.085	13	2	31.4	7.36	209.1	47.1	89.9	54
568	O15042	U2 snRNP-associated SURP motif-containing protein [OS=Homo sapien:	↓	6.071	3	3	118.2	8.47	130.3	103.7	86	80.1
569	Q8NBJ4	Golgi membrane protein 1 [OS=Homo sapiens]	↓	5.979	6	2	45.3	4.97	134	66.4	106	93.6
570	Q14166	Tubulin--tyrosine ligase-like protein 12 [OS=Homo sapiens]	↓	5.956	3	2	74.4	5.53	147.7	81.7	107.4	63.3
571	P56545	c-terminal-binding protein 2 [OS=Homo sapiens]	↓	5.931	4	2	48.9	6.95	132.1	111.1	99	57.9
572	Q9H9H4	Vacuolar protein sorting-associated protein 37B [OS=Homo sapiens]	↑	5.786	10	2	31.3	7.34	36.9	111.6	75.8	175.7
573	P51580	Thiopurine S-methyltransferase [OS=Homo sapiens]	↓	5.765	9	2	28.2	6.23	168.1	105.2	102.6	24.1
574	Q93034	Cullin-5 [OS=Homo sapiens]	↓	5.42	5	2	90.9	7.94	131.1	127.5	116.2	25.3
575	P02649	Apolipoprotein E [OS=Homo sapiens]	↑	5.333	5	2	36.1	5.73	75.6	190.8	58.1	75.5
576	P23141	Liver carboxylesterase 1 [OS=Homo sapiens]	↑	5.214	4	2	62.5	6.6	66	137	96	101
577	Q15746-1	Myosin light chain kinase, smooth muscle [OS=Homo sapiens]	↓	5.052	1	2	210.6	6.15	134.6	104.2	101.6	59.6
578	P09669	Cytochrome c oxidase subunit 6C [OS=Homo sapiens]	↓	4.745	20	2	8.8	10.39	182.9	67.2	85.4	64.5
579	P02452	Collagen alpha-1(I) chain [OS=Homo sapiens]	↑	3.976	2	2	138.9	5.8	49.6	152.9	110.7	86.8
580	P28482	mitogen-activated protein kinase 1 [OS=Homo sapiens]	↓	3.921	9	2	41.4	6.98	146	96.1	91.4	66.4
581	Q15031	Probable Leucine--tRNA ligase, mitochondrial [OS=Homo sapiens]	↓	3.915	5	2	101.9	8.22	141	73.5	108.1	77.4
582	Q9Y6V0-1	Protein piccolo [OS=Homo sapiens]	↑	2.807	1	2	552.9	6.51	67.6	105	129.5	97.9
